# Supplementary material for: Technical and biological sources of unreliability of Infinium probes on Illumina methylation microarrays
Source: Clin Epigenetics. 2024 Sep 18;16:131. doi: 10.1186/s13148-024-01739-2 (PMC11409515; doi:10.1186/s13148-024-01739-2)
Supplement: Supplementary file 1 — Additional file 1 Supplementary Figures S1–S40. [file 13148_2024_1739_MOESM1_ESM.pdf]

Additional file 1: Supplementary Figures

Technical and biological sources of unreliability of the Infinium probes on the Illumina methylation microarrays

Tatiana Nazarenko[1,2,3], Charlotte D. Vavourakis[1,2], Allison Jones[3], Iona Evans[3], Lena Schreiberhuber[1,2], Christine Kastner[1,2], Isma Ishaq-Parveen[1,2], Elisa Redl[1,2], Anthony W. Watson[4], Kirsten Brandt[4], Clive Carter[5], Alexey Zaikin[3], Chiara Herzog[1,2*] and Martin Widschwendter[1,2,3*]

[1] Research Institute for Biomedical Aging Research, Universität Innsbruck, Innsbruck, 6020, Austria.

[2] European Translational Oncology Prevention and Screening (EUTOPS) Institute, Milser Str. 10, Hall in Tirol, 6060, Austria.

[3] Department of Women's Cancer, UCL EGA Institute for Women's Health, University College London, Medical School Building, Room 340, 74 Huntley Street, London, WC1E 6AU, UK.

[4] Human Nutrition Research Centre, Population Health Sciences Institute, Newcastle University, Newcastle upon Tyne, NE1 7RU, UK.

[5] Transplant and Cellular Immunology Laboratories, Leeds Teaching Hospital NHS Trust, St James's University Hospital, Leeds, LS9 7TF, UK.

* Correspondence: Email: Chiara.Herzog@uibk.ac.at or Martin.Widschwendter@uibk.ac.at

a

				TRUE FRACTIONS (when **C** in 0-position on 1 or 2 chromosomes)		FALSE FRACTIONS (when **SNP** in 0-position on 1 or 2 chromosomes)		
**TYPE**	**ALTERNATIVE**	**POSSIBLE VARIANTS**	**PROBABILITY OF VARIANTS**	**GREEN FRACTION**	**RED FRACTION**	**FALSE GREEN FRACTION**	**FALSE RED FRACTION**	**GREEN/(GREEN + RED)**
**NON SNP_0**	**[C]**	CC	P=1	X	1-X	0	0	BETA = X
**SNP_0**	**[C/G]**	CC	$P = P_C P_C$	X	1-X	0	0	BETA = X
		CG or GC	$P = 2P_C P_G$	0.5X	0.5(1-X)	0.5	0	BETA =0.5X + 0.5
		GG	$P = P_G P_G$	0	0	1	0	BETA = 1
	**[C/T]**	CC	$P = P_C P_C$	X	1-X	0	0	BETA = X
		CT or TC	$P = 2P_C P_T$	0.5X	0.5(1-X)	0	0.5	BETA =0.5X
		TT	$P = P_T P_T$	0	0	0	1	BETA = 0
	**[C/A]**	CC	$P = P_C P_C$	X	1-X	0	0	BETA = X
		CA or AC	$P = 2P_C P_A$	0.5X	0.5(1-X)	0	0.5	BETA =0.5X
		AA	$P = P_A P_A$	0	0	0	1	BETA = 0

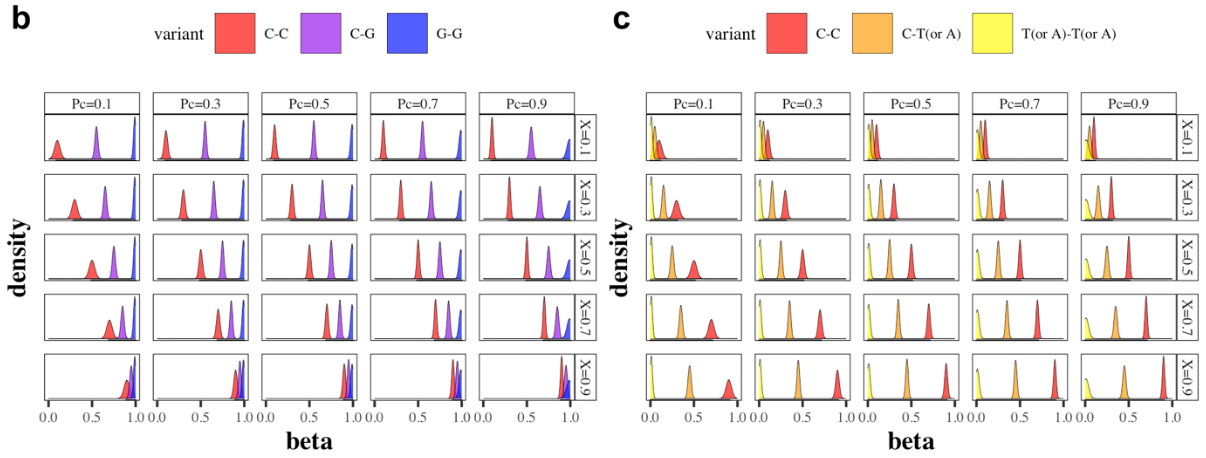

Figure S1. Formation of tri-modal distributions of beta-values in the presence of a SNP in 0-position (C in the CG pair). (a) The table shows how, in the case of the presence of SNP in the 0-position (in C base of target CG pair), depending on the nucleotide (G or T/A), a trimodal distribution of beta-values arises, where each mode is represented by carriers of a certain variant. For example, in the case when C base in the CG pair can be replaced by G in population, then the entire population is divided into three groups of carriers of different variants (CC - C base on both chromosomes; CG or GC - when C (or G) base is present only on one chromosome; GG - G base on both chromosomes). Carriers of the CC variant will receive true green and red signals (let their average beta value be X). Carriers of the CG or GC variant will receive true green and red methylation signals from one chromosome and a false green signal from the other (which will cause their beta-values to shift to the right, towards increased methylation and the average signal will be approximately 0.5X + 0.5); Carriers of the GG variant will get false green signal from both chromosomes and the average beta-values will be close to 1). Similarly, trimodal distributions of beta-values are formed if the C base in CG pair can be replaced in the population by T or A. In this case, for carriers of T or A bases (on one or both chromosomes) a false red signal will accumulate, and the modes of their beta-values will shift to the left (will be lower) than the mode of carriers of the CC variant. The mode density in such tri-modal distributions is determined by the probability of the variant, defined through the probability of the SNP or the probability of C. Simulation of tri-modal distributions for both situations: when C base in the target CG pair can be replaced by G base **(b)** or T/A base **(c)**, depending on the probability C and on X - true beta signal (from carriers of CC variant).

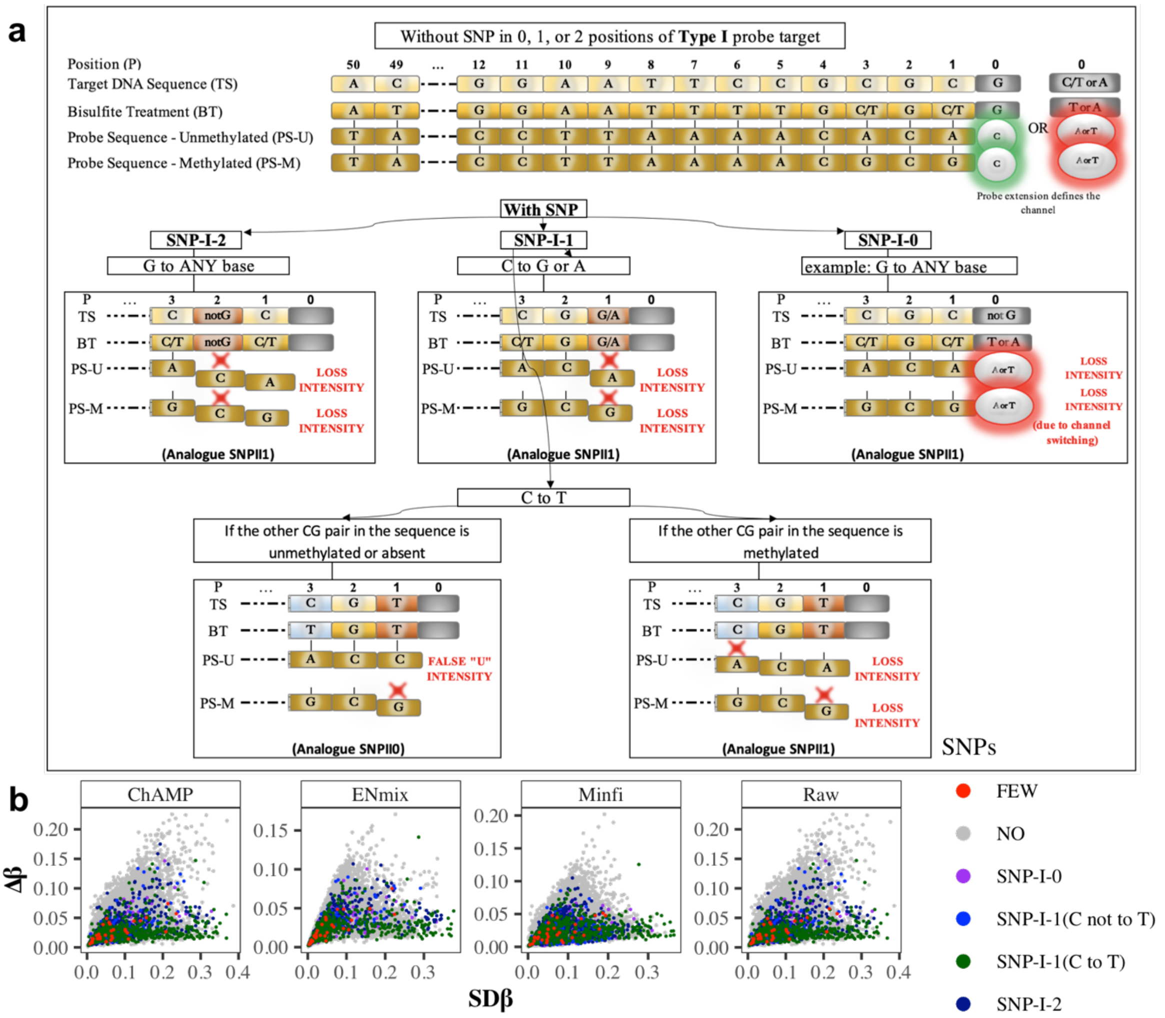

Figure S2. Variability associated with genetic variants (Type I probes). (a) Three types of SNPs and their impact on signal: SNPs in position 0 (SNP-I-0) can be result a loss of signal due to channel switching in case if original G base has as a variant any other base (as shown on example), or if original C/T/A base has as a variant G base; SNPs in position 2 (SNP-I-2) and 1 when C base base has as a variant G or A (SNP-I-1(C not to T)), or when C has a variant T (SNP-I-1(C to T)), and other CG pairs in the sequences are methylated result in a loss of signal due to the fact that the bisulfite sequence is no longer complementary to the U and M probes; SNPs in position 1 when C base has a variant T (SNP-I-1(C to T)) and other CG pairs in the sequences are unmethylated or absent result in false U signals. **(b)** SNPs in position 2,1 and 0 demonstrate high variability in the population at visit 1 (SD β) and SNP-I-0, SNP-I-2, SNP-I-1(C not to T) and part of SNP-I-1(C to T) show the same effect as SNP-II-1 probes (i.e. demonstrate high variability over time (Δ β)), and other part of SNP-I-1(C to T) shows the same effect as SNP-II-0 probes.

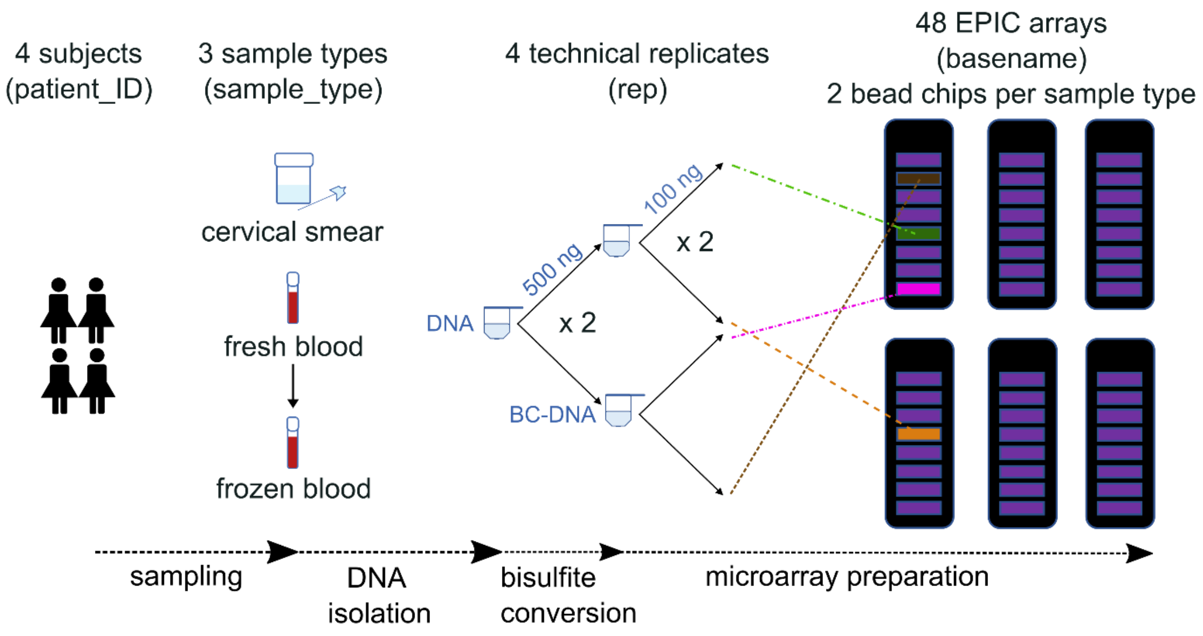

Figure S3. Experimental design repeatability set. Whole blood and cervical smear samples were collected from four female, healthy subjects. DNA from the blood was isolated either directly (fresh blood) or from the left-over sample that was kept frozen for prolonged storage. Four technical replicates were created from each DNA mixture: two times 500 ng from the same mixture was bisulfite converted (BC), and from each BC-DNA mixture two times 100 ng was prepared for hybridization to the microarrays. For each sample type, i.e. cervical smear, fresh blood or frozen blood, the positions of the samples and replicates were randomized across two bead chips.

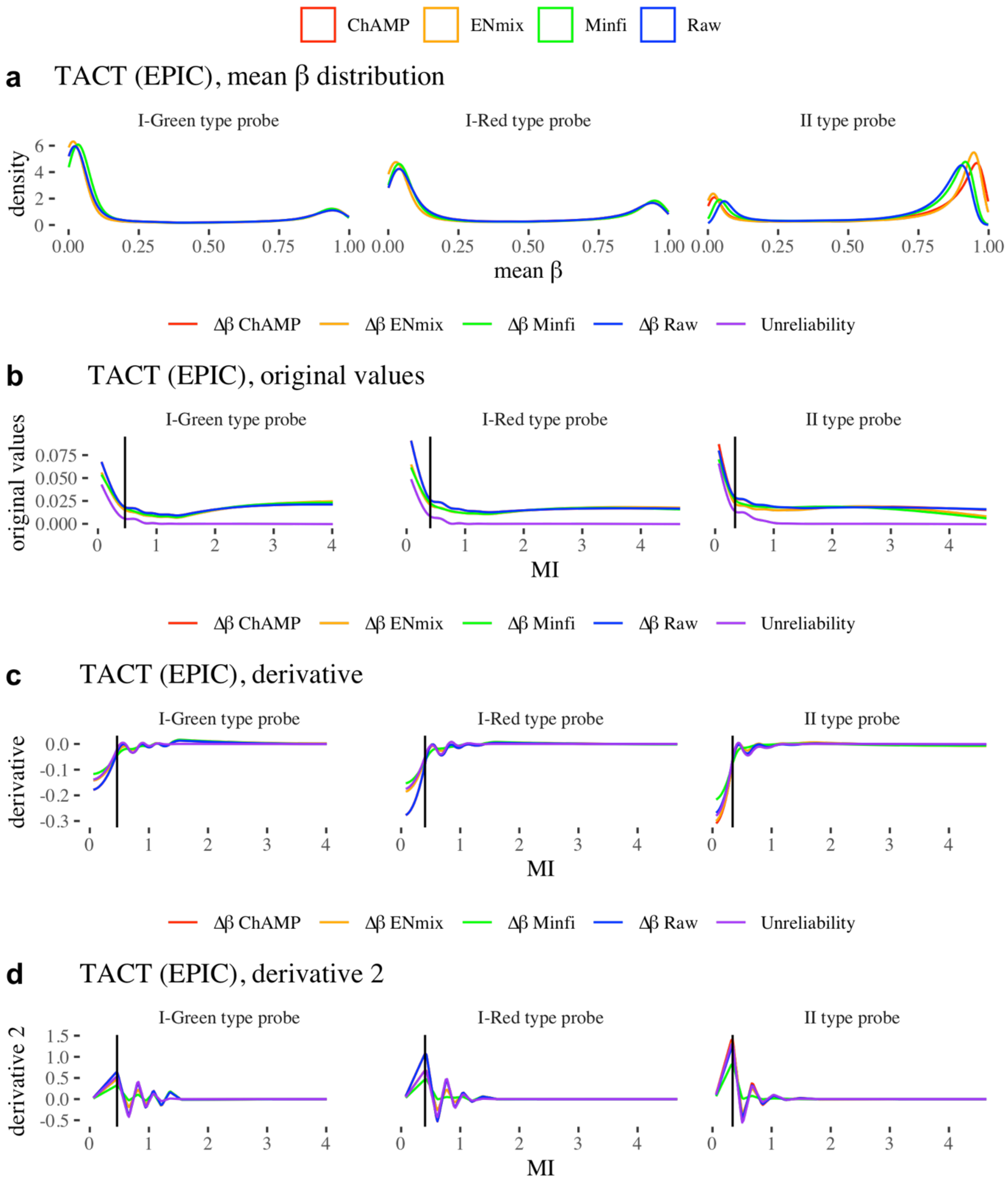

Figure S4. **(a)** Distribution of probes mean β (averaged by samples). **(b)** Averaged, absolute methylation differences in methylation values between repeated samples (Δβ) and associated unreliability scores as a function of MI in the longitudinal TACT dataset with ('ChAMP', 'Enmix', 'Minfi') and without ('Raw') different normalization methods are plotted for each probe type/color, and **(c)** first and **(d)** second derivatives thereof.

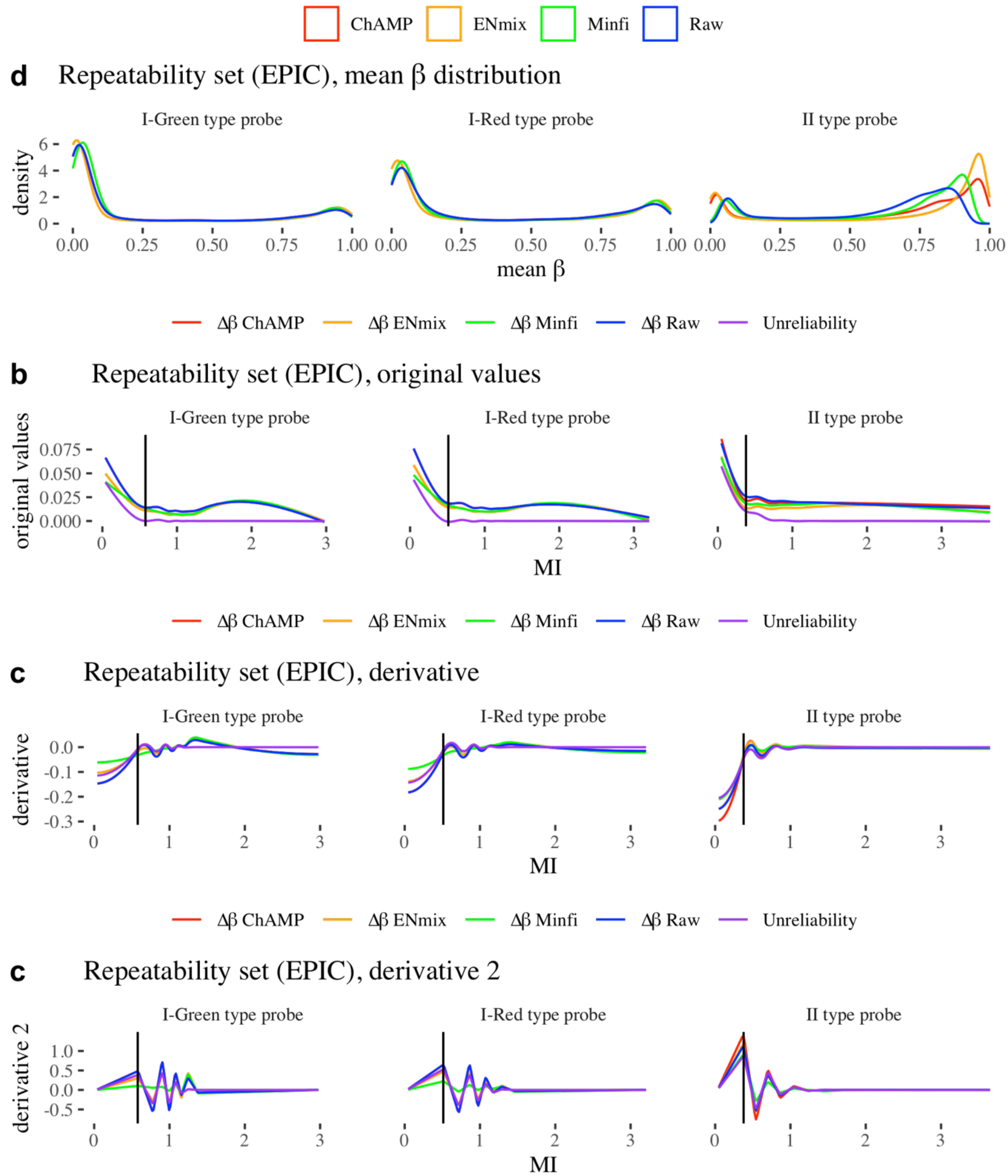

Figure S5. (a) Distribution of probes mean β (averaged by samples). **(b)** Averaged, absolute methylation differences in methylation values between technical replicates (Δβ) and associated unreliability scores as a function of MI in the longitudinal Repeatability dataset with ('ChAMP', 'Enmix', 'Minfi') and without ('Raw') different normalization methods are plotted for each probe type/color, and **(c)** first and **(d)** second derivatives thereof.

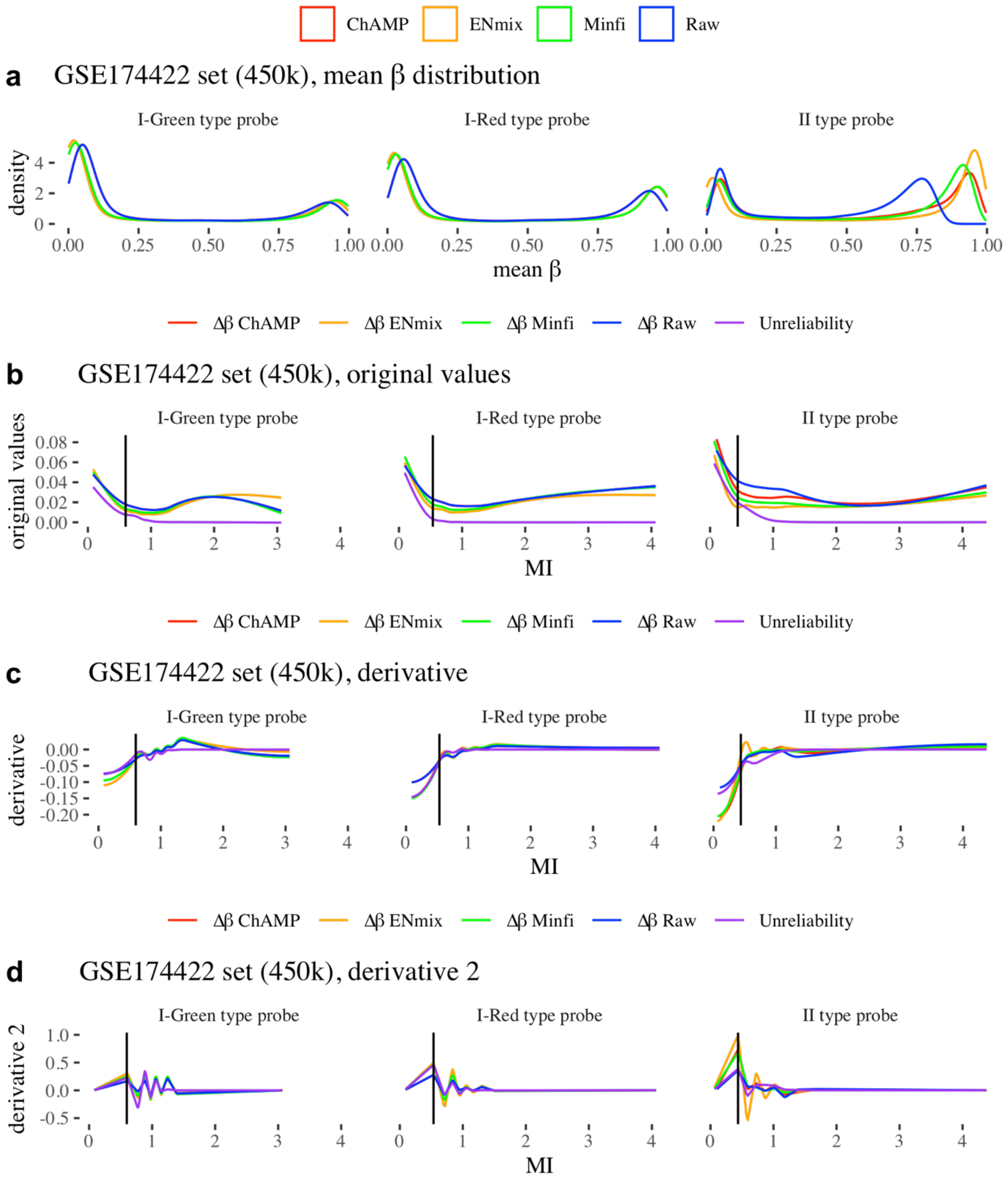

Figure S6. (a) Distribution of probes mean β (averaged by samples). **(b)** Averaged, absolute methylation differences in methylation values between technical replicates (Δβ) and associated unreliability scores as a function of MI in the GSE174422 dataset with ('ChAMP', 'Enmix', 'Minfi') and without ('Raw') different normalization methods are plotted for each probe type/color, and **(c)** first and **(d)** second derivatives thereof.

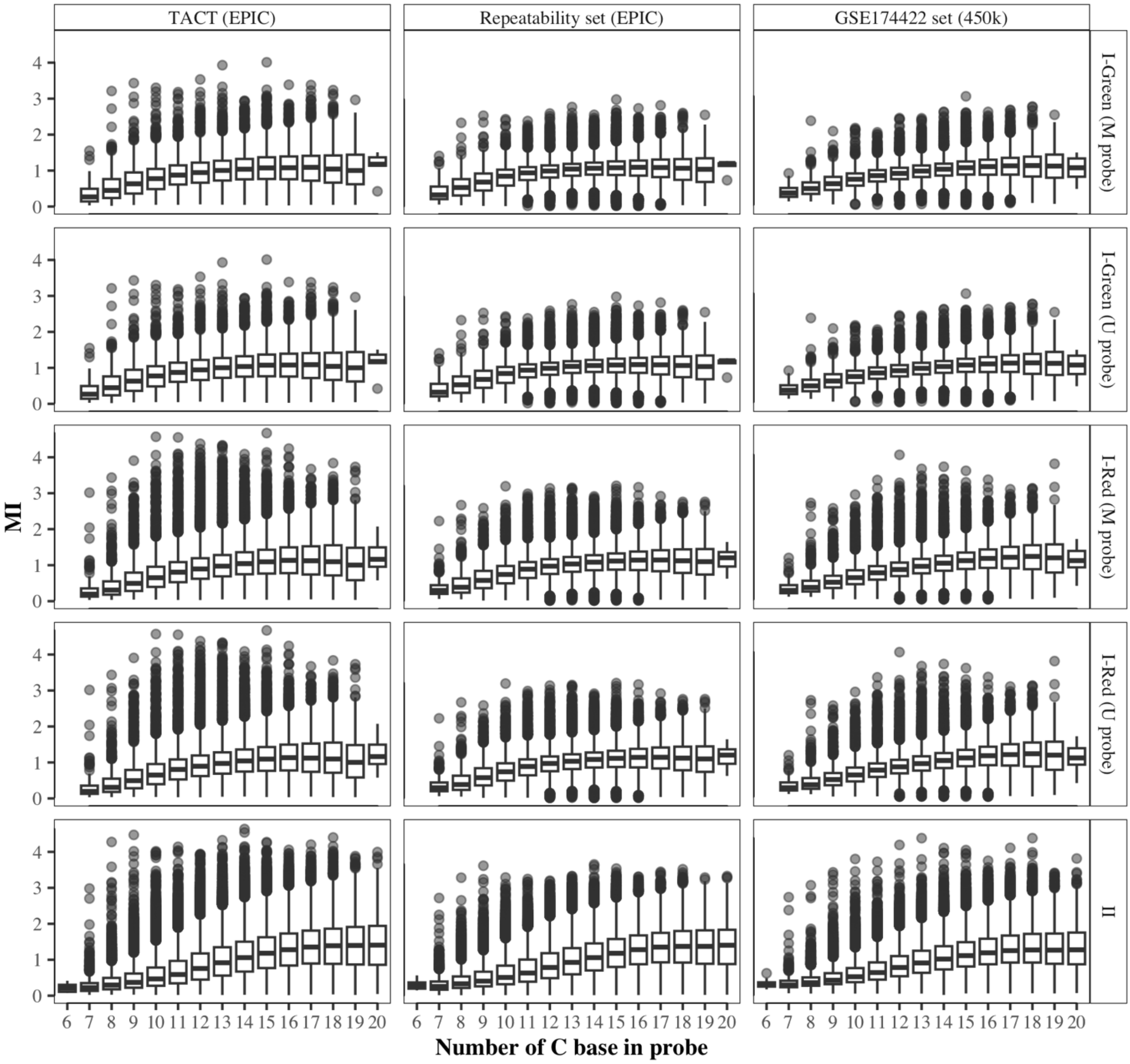

Figure S7. Dependence of mean signal intensity (MI) on C content of type I probes in different DNAme data sets.

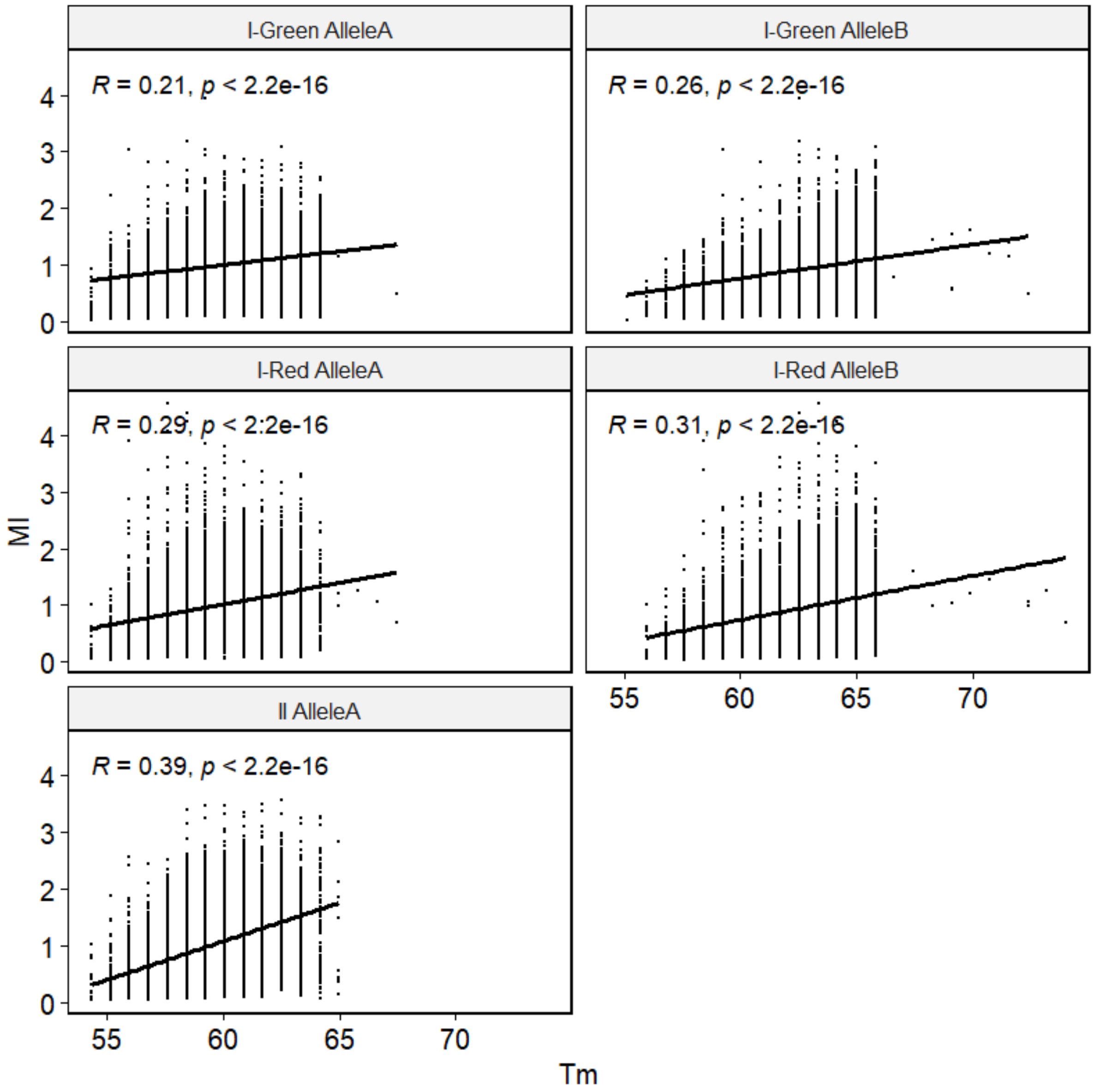

Figure S8. Estimated probe melting temperature (Tm) of EPICv1 probes versus MI scores across first visit samples from 53 female participants in the TACT study. To avoid overplotting, only a random subset of 10,000 CpG sites across chromosomes 1-23 for each probe type and methylated/unmethylated allele are shown.

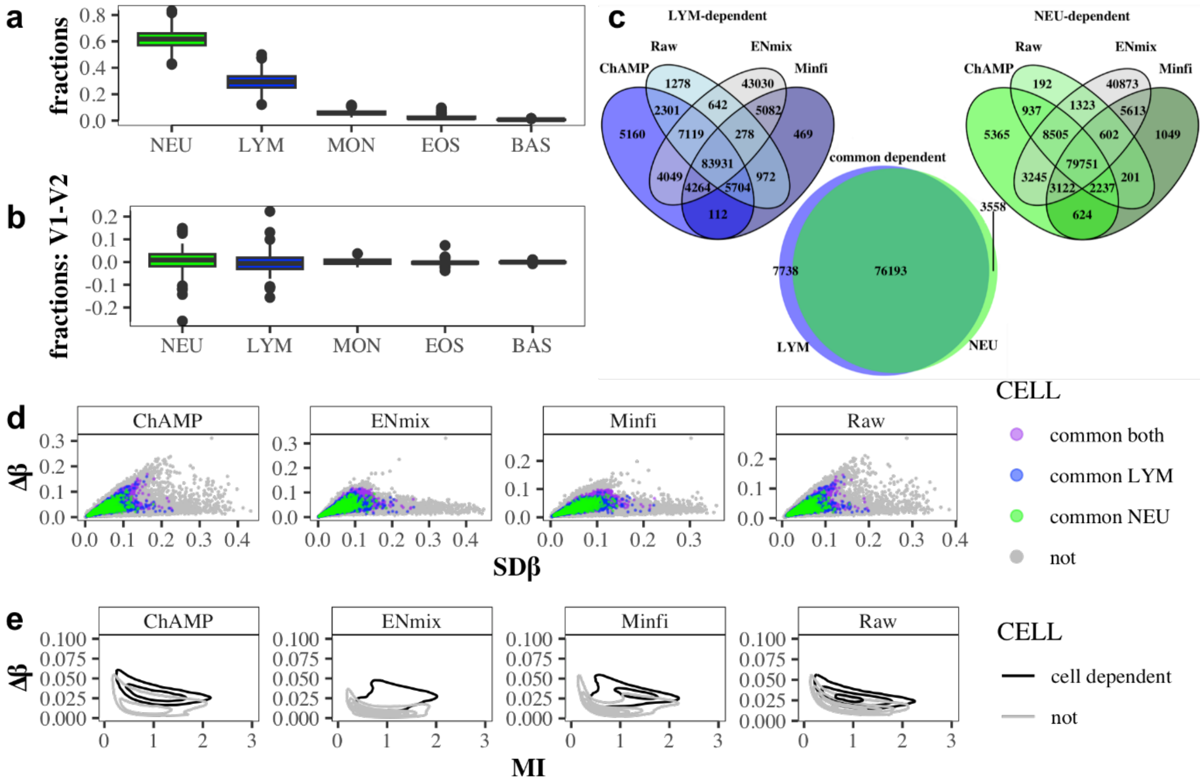

Figure S9. The effect of cell subtype composition on DNAme variability over time (example for type II probes) (a) Distribution of cell subtypes fractions in the samples (both visits together); **(b)** Difference in cell subtype fractions for each patient between the two visits; NEU = neutrophils, LYM = lymphocytes, MON = monocytes, EOS = eosinophils, BAS = basophils. **(c)** Venn Diagram of cell subtype-dependent probes for ChAMP, minfi, and the raw β generation; **(d)** The variability of the probes dependent on one of the cell subtypes fraction, where 'common LYM' – probes significantly detected as lymphocyte-fraction dependent probes for all four pipelines, 'common NEU' – probes significantly detected as neutrophils-fraction dependent probes for all four pipelines, 'common both' – probes significantly detected as neutrophils-fraction dependent and lymphocyte-fraction dependent probes for all four pipelines; **(e)** 2D density plot of variation over time versus MI which shows that cell subtype-dependent probes exhibit high variability in time (which may be interpreted as variability depending on the cellular composition).

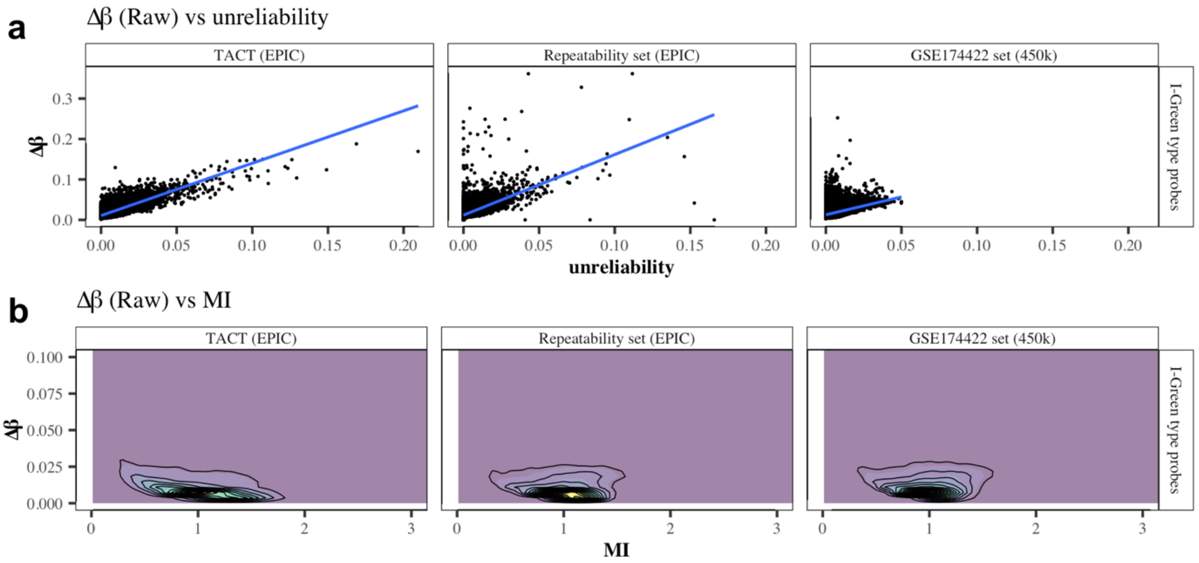

Figure S10. Association of type I-Green probe variability (Δβ) with **(a)** the unreliability score and **(b)** the mean intensity (MI) in the TACT study (longitudinal paired blood samples), the Repeatability set (n=4x4x4 technical replicates for fresh blood, frozen blood and cervical smear samples) and GSE174422 (n=2x128 technical replicates for blood samples).

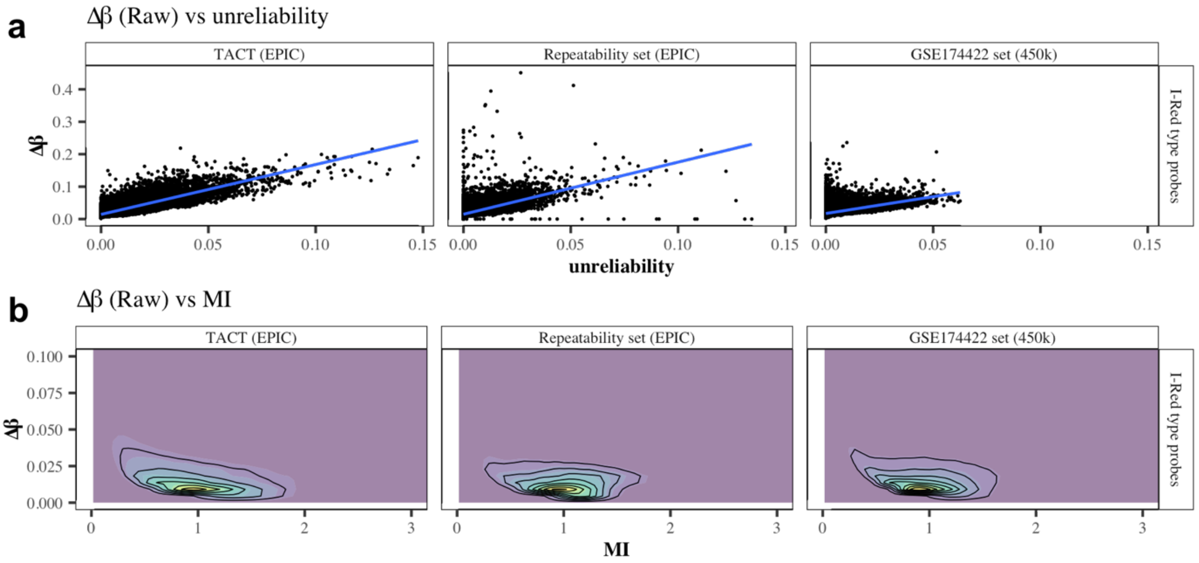

Figure S11. Association of type I-Red probe variability (Δβ) with **(a)** the unreliability score and **(b)** the mean intensity (MI) in the TACT study (longitudinal paired blood samples), the Repeatability set (n=4x4x4 technical replicates for fresh blood, frozen blood and cervical smear samples) and GSE174422 (n=2x128 technical replicates for blood samples).

a

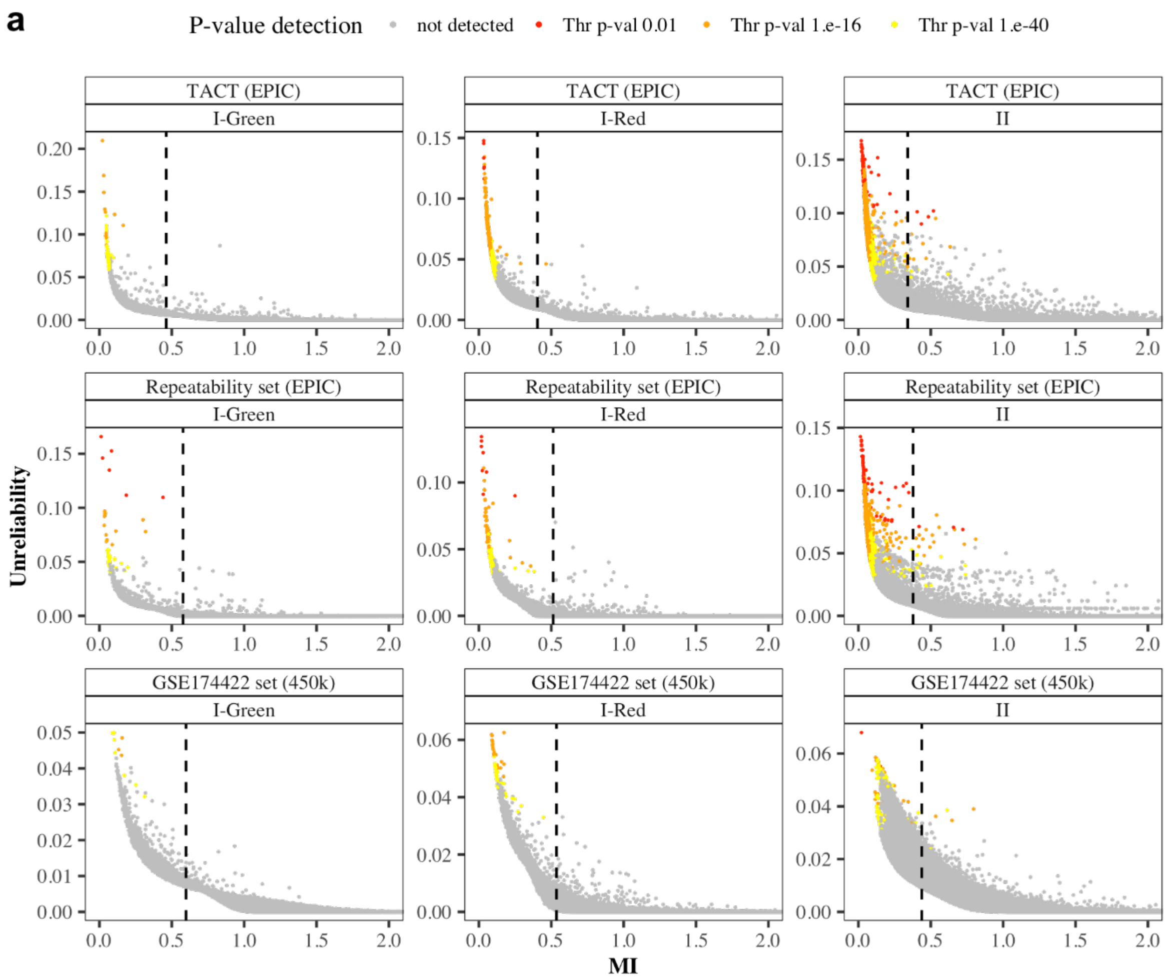

b

Dataset	I-Green						I-Red						II					
	#probes	#probes (without SNPs and X/Y CHR)	#probes detected with p-val 0.01	#probes detected with p-val 1.e-16	#probes detected with p-val 1.e-40	#probes detected with Unreliability	#probes	#probes (without SNPs and X/Y CHR)	#probes detected with p-val 0.01	#probes detected with p-val 1.e-16	#probes detected with p-val 1.e-40	#probes detected with Unreliability	#probes	#probes (without SNPs and X/Y CHR)	#probes detected with p-val 0.01	#probes detected with p-val 1.e-16	#probes detected with p-val 1.e-40	#probes detected with Unreliability
TACT (EPIC)	49939	48072 (100%)	0 (0%)	13 (0.03%)	56 (0.12%)	**5684 (11.82%)**	92198	88687 (100%)	6 (0.01%)	229 (0.26%)	764 (0.86%)	**9967 (11.24%)**	723722	697357 (100%)	76 (0.01%)	2291 (0.33%)	8042 (1.15%)	**87889 (12.6%)**
Repeatability set (EPIC)	49939	48072 (100%)	6 (0.01%)	18 (0.04%)	47 (0.1%)	**5481 (11.4%)**	92198	88687 (100%)	8 (0.01%)	59 (0.07%)	181 (0.2%)	**10186 (11.49%)**	723659	697297 (100%)	61 (0.01%)	899 (0.13%)	3486 (0.5%)	**85016 (12.19%)**
GSE174422 set (450k)	46289	44588 (100%)	0 (0%)	3 (0.01%)	11 (0.02%)	**4925 (11.05%)**	89187	85930 (100%)	0 (0%)	18 (0.02%)	62 (0.07%)	**10199 (11.87%)**	350036	335980 (100%)	2 (0%)	33 (0.01%)	162 (0.05%)	**41796 (12.44%)**

Figure S12. (a) Dependence of probe unreliability on MI, highlighting probes which are detected using the p-value method at different threshold stringency. **(b)** Number of probes removed by the respective methods.

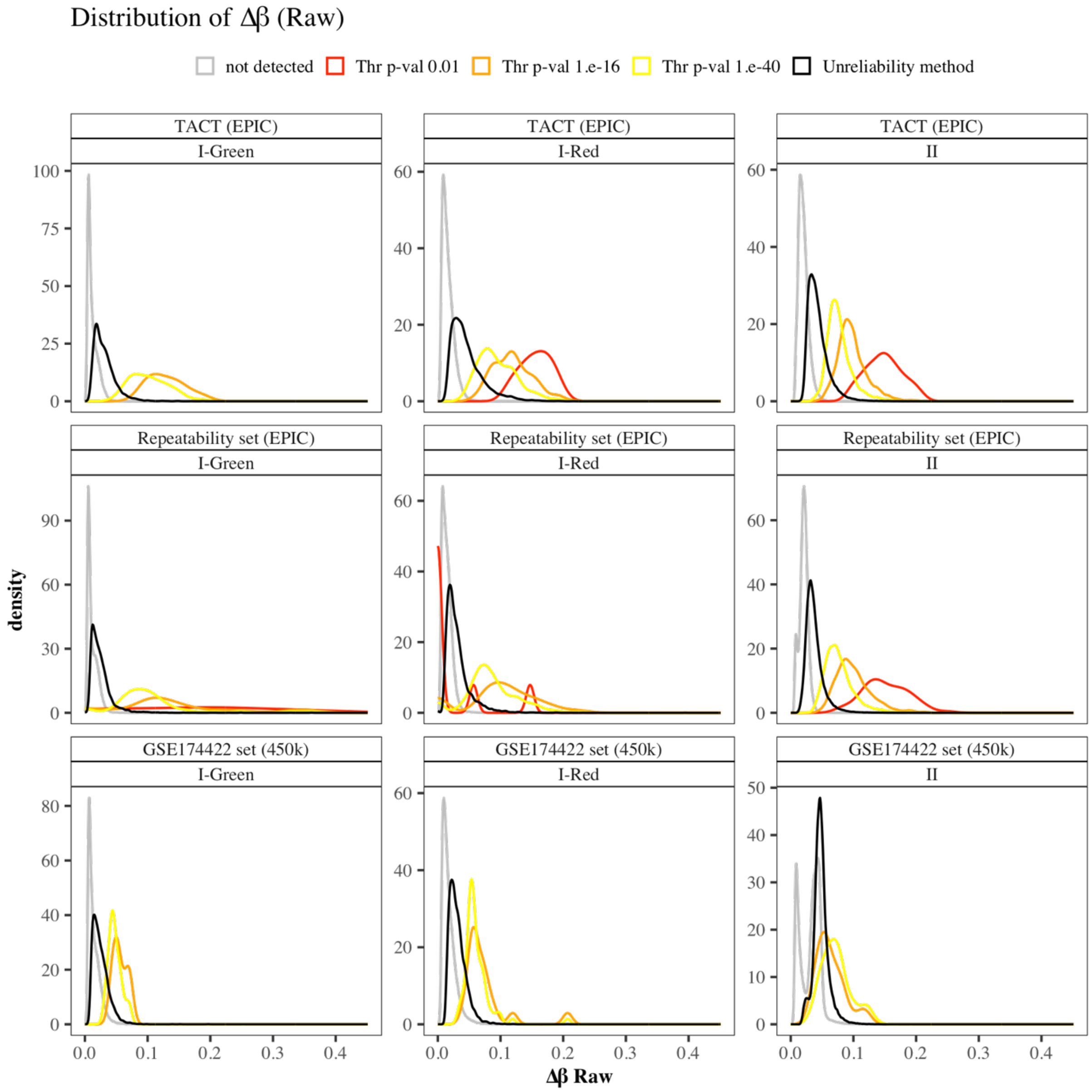

Figure S13. Distribution of the averaged, absolute methylation differences in methylation values between repeated samples and technical replicates (Δβ) for all probes (grey) or those removed by the p-value detection method at different threshold settings (red, orange, yellow) and the Unreliability method (black). β-values were not normalized.

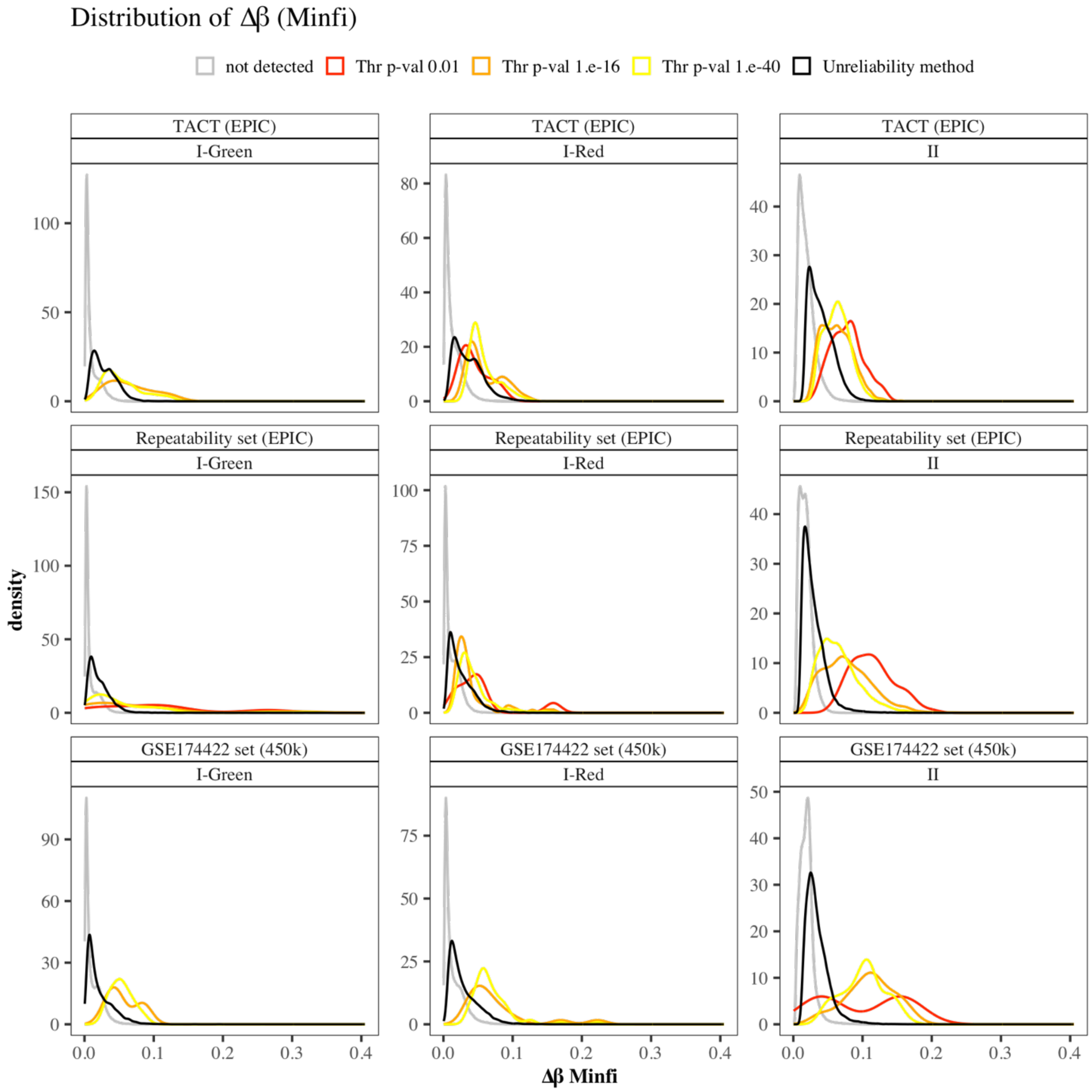

Figure S14. Distribution of the averaged, absolute methylation differences in methylation values between repeated samples and technical replicates (Δβ) for all probes (grey) or those removed by the p-value detection method at different threshold settings (red, orange, yellow) and the Unreliability method (black). β-values were normalized using the preprocessFunnorm() function in the R package *minfi*.

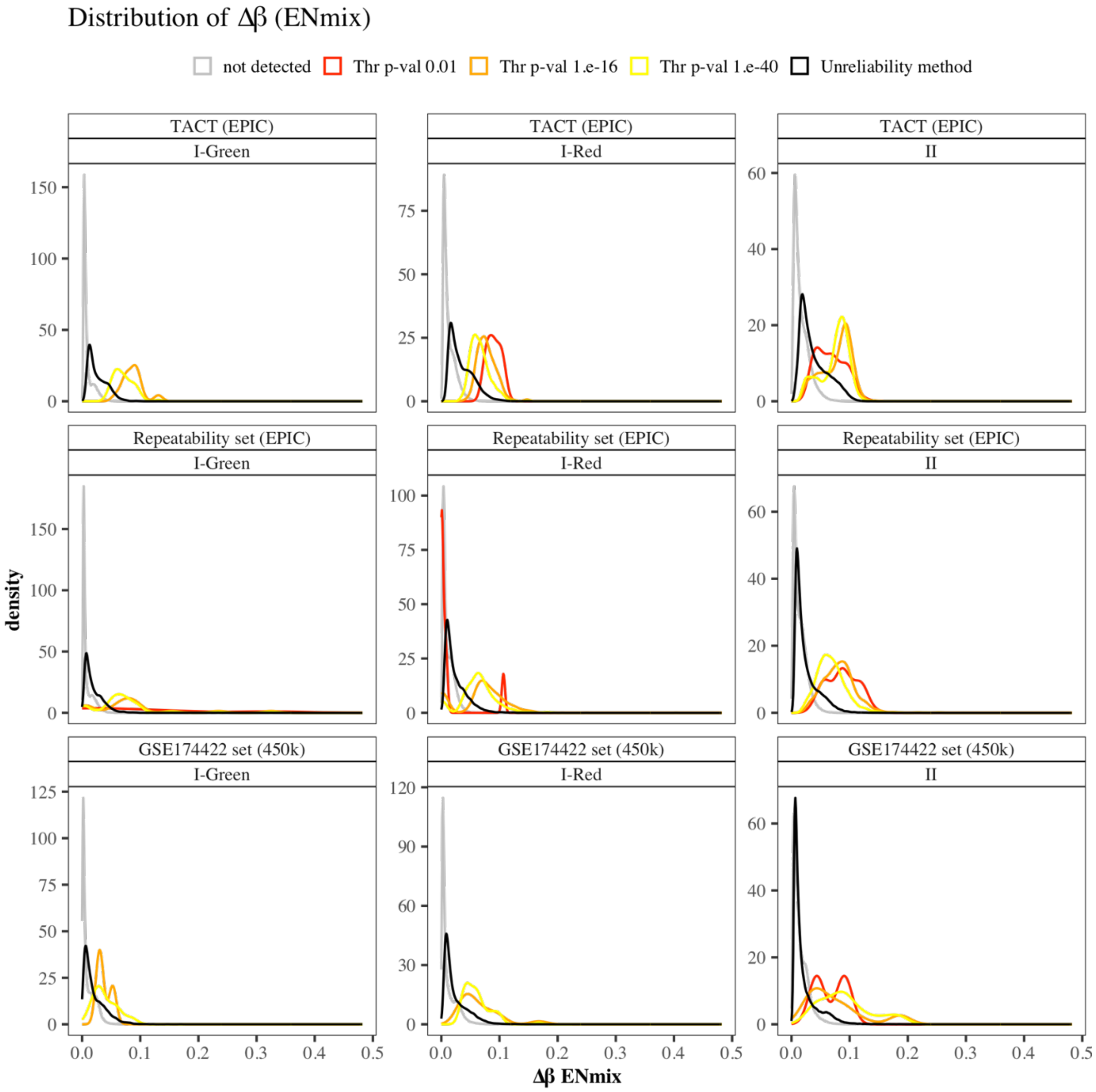

Figure S15. Distribution of the averaged, absolute methylation differences in methylation values between repeated samples and technical replicates (Δβ) for all probes (grey) or those removed by the p-value detection method at different threshold settings (red, orange, yellow) and the Unreliability method (black). β-values were normalized using the R package *ENmix*.

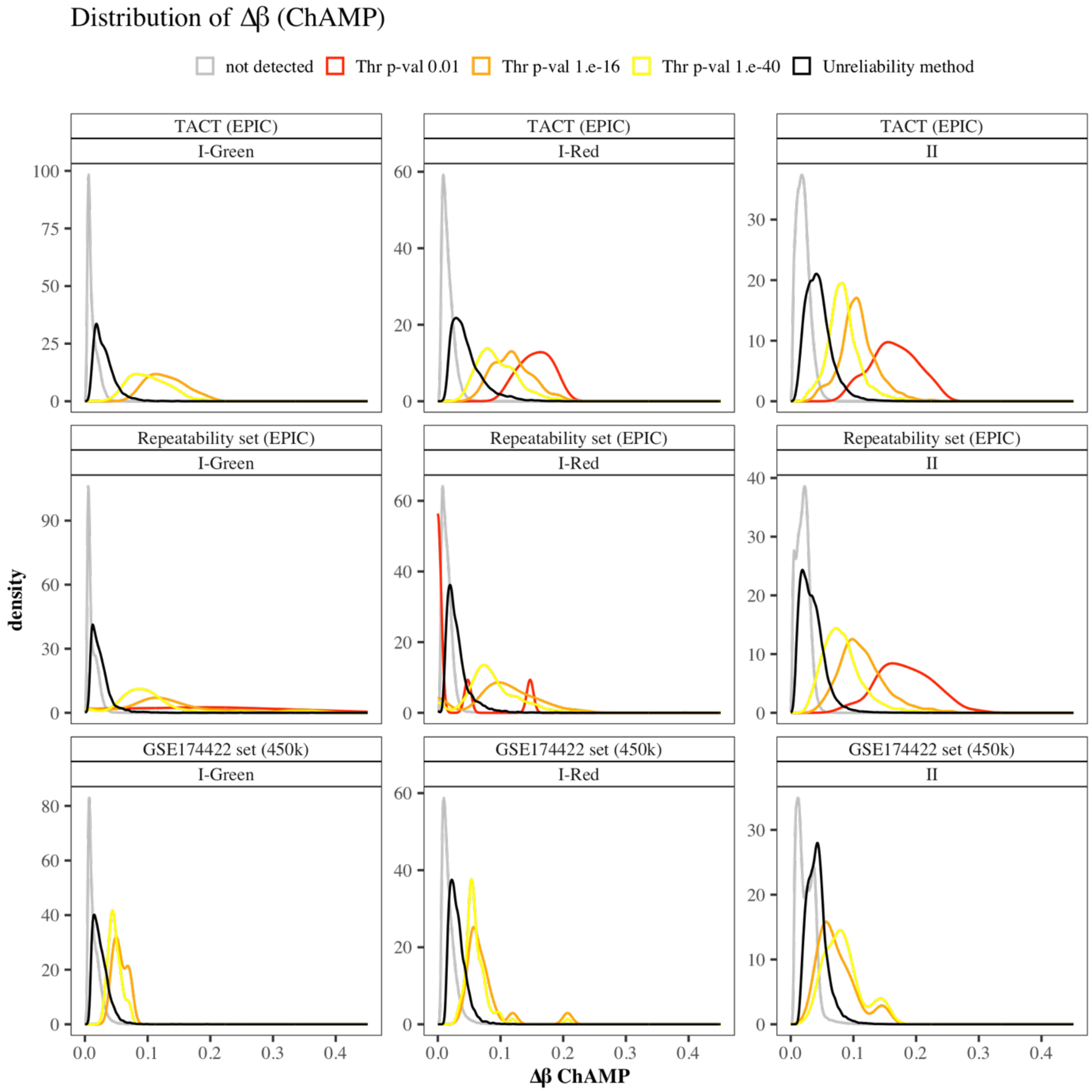

Figure S16. Distribution of the averaged, absolute methylation differences in methylation values between repeated samples and technical replicates (Δβ) for all probes (grey) or those removed by the p-value detection method at different threshold settings (red, orange, yellow) and the Unreliability method (black). β-values were normalized using the R package *ChAMP*.

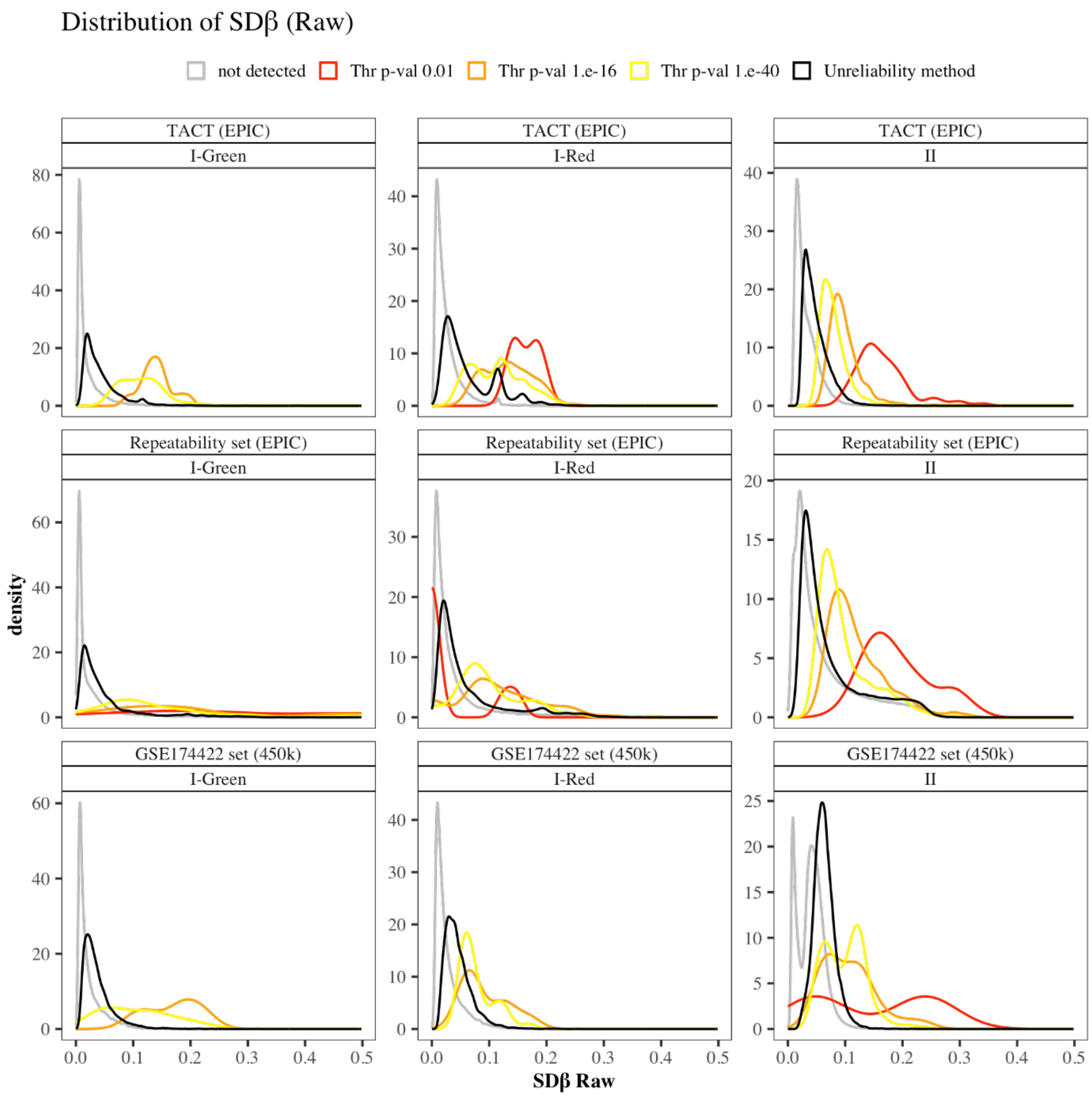

Figure S17. Distribution of the standard deviations obtained for methylation values (SDβ) for all probes (grey) or those removed by the p-value detection method at different threshold settings (red, orange, yellow) and the Unreliability method (black). β-values were not normalized.

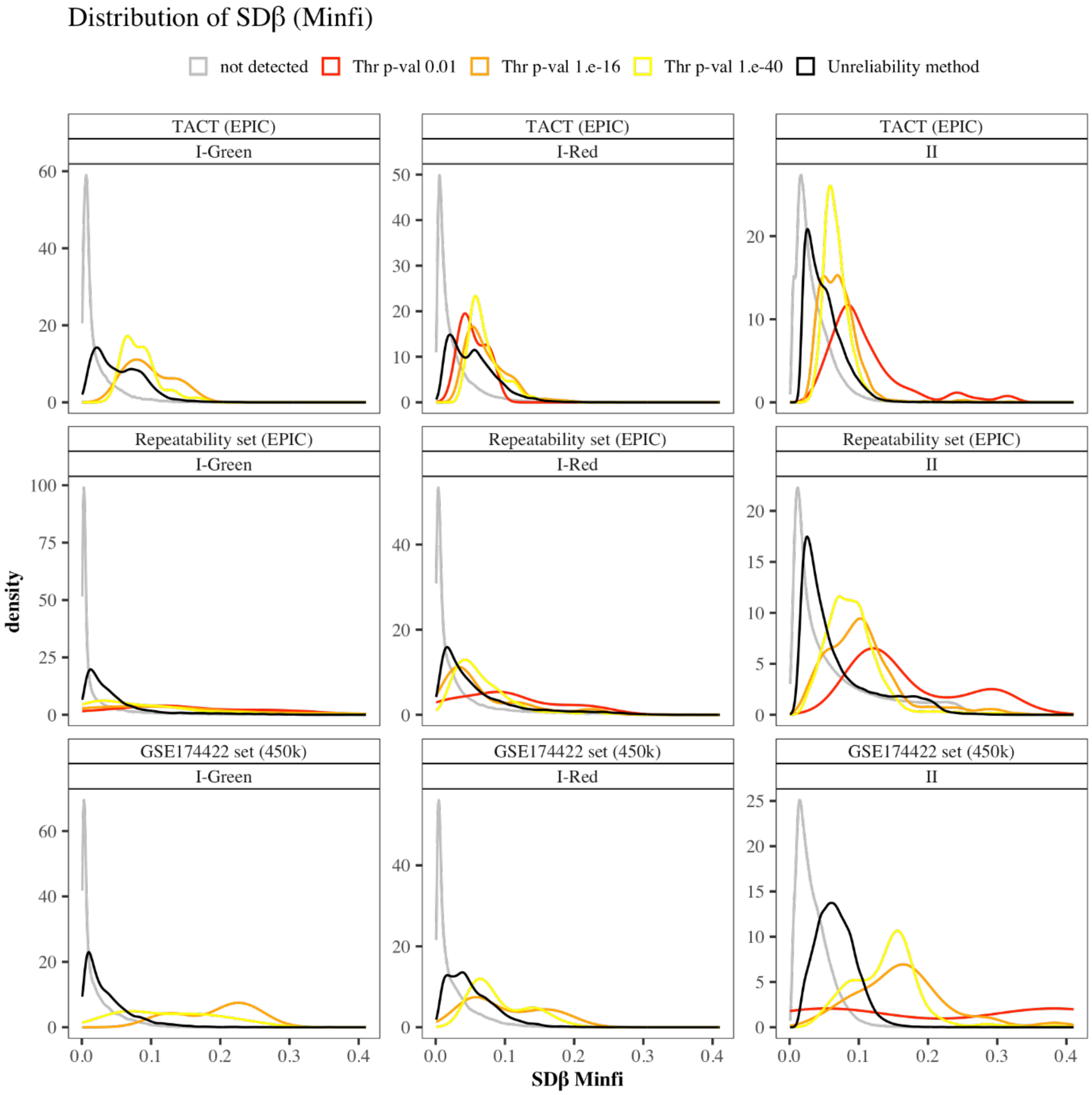

Figure S18. Distribution of the standard deviations obtained for methylation values (SDβ) for all probes (grey) or those removed by the p-value detection method at different threshold settings (red, orange, yellow) and the Unreliability method (black). β-values were normalized using the preprocessFunnorm() function in the R package *minfi*.

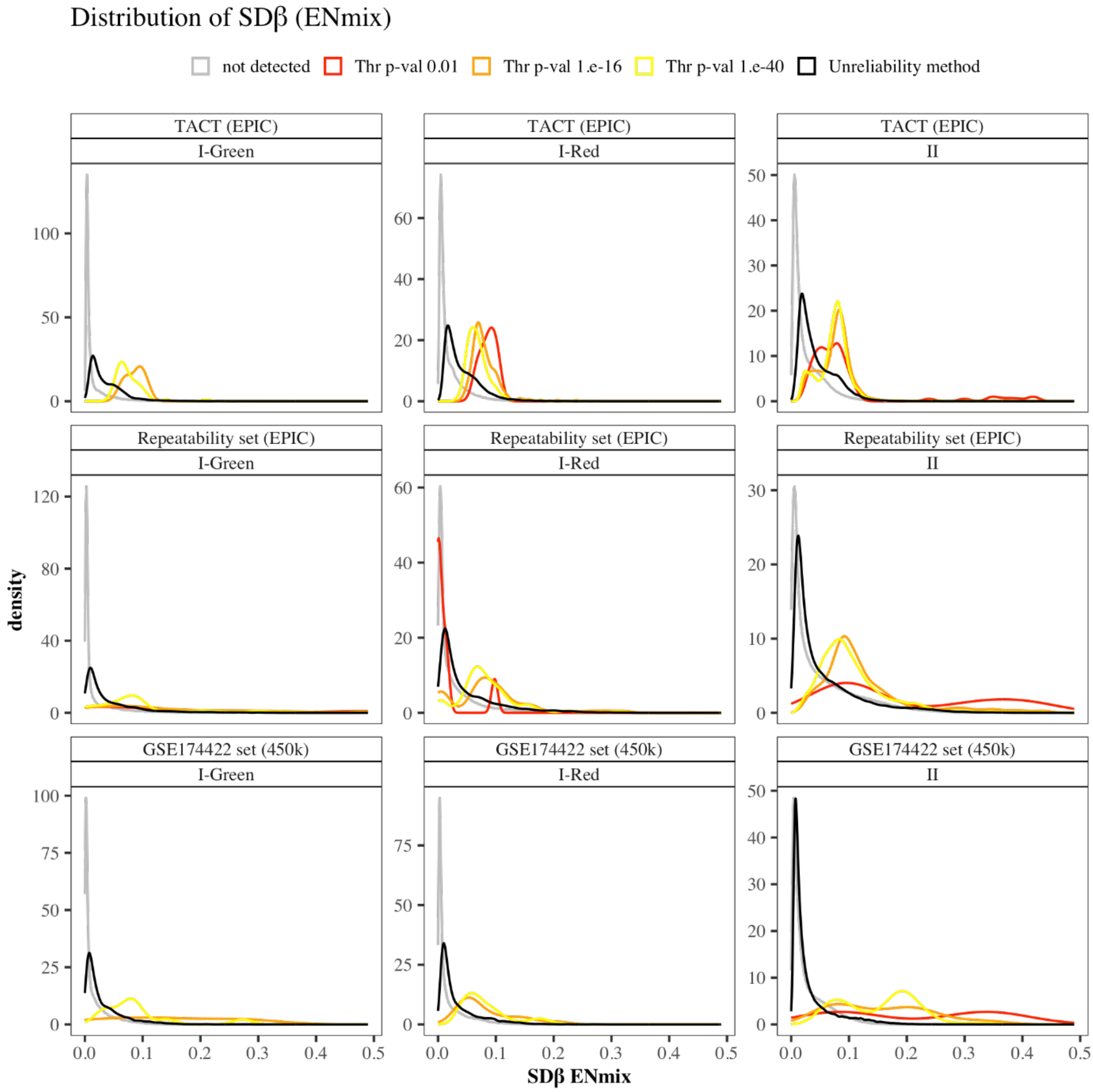

Figure S19. Distribution of the standard deviations obtained for methylation values (SDβ) for all probes (grey) or those removed by the p-value detection method at different threshold settings (red, orange, yellow) and the Unreliability method (black). β-values were normalized using the R package *ENmix*.

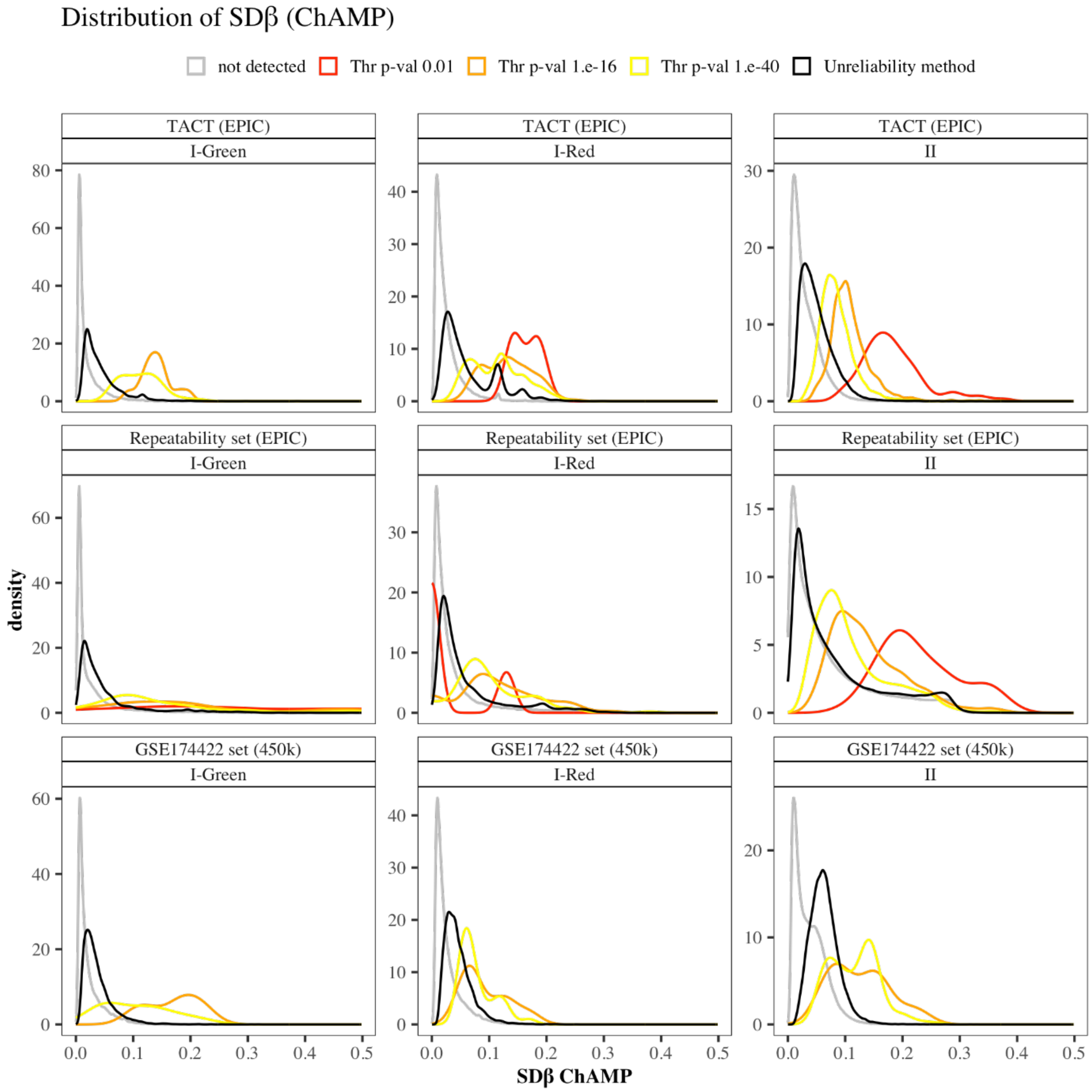

Figure S20. Distribution of the standard deviations obtained for methylation values (SDβ) for all probes (grey) or those removed by the p-value detection method at different threshold settings (red, orange, yellow) and the Unreliability method (black). β-values were normalized using the R package *ChAMP*.

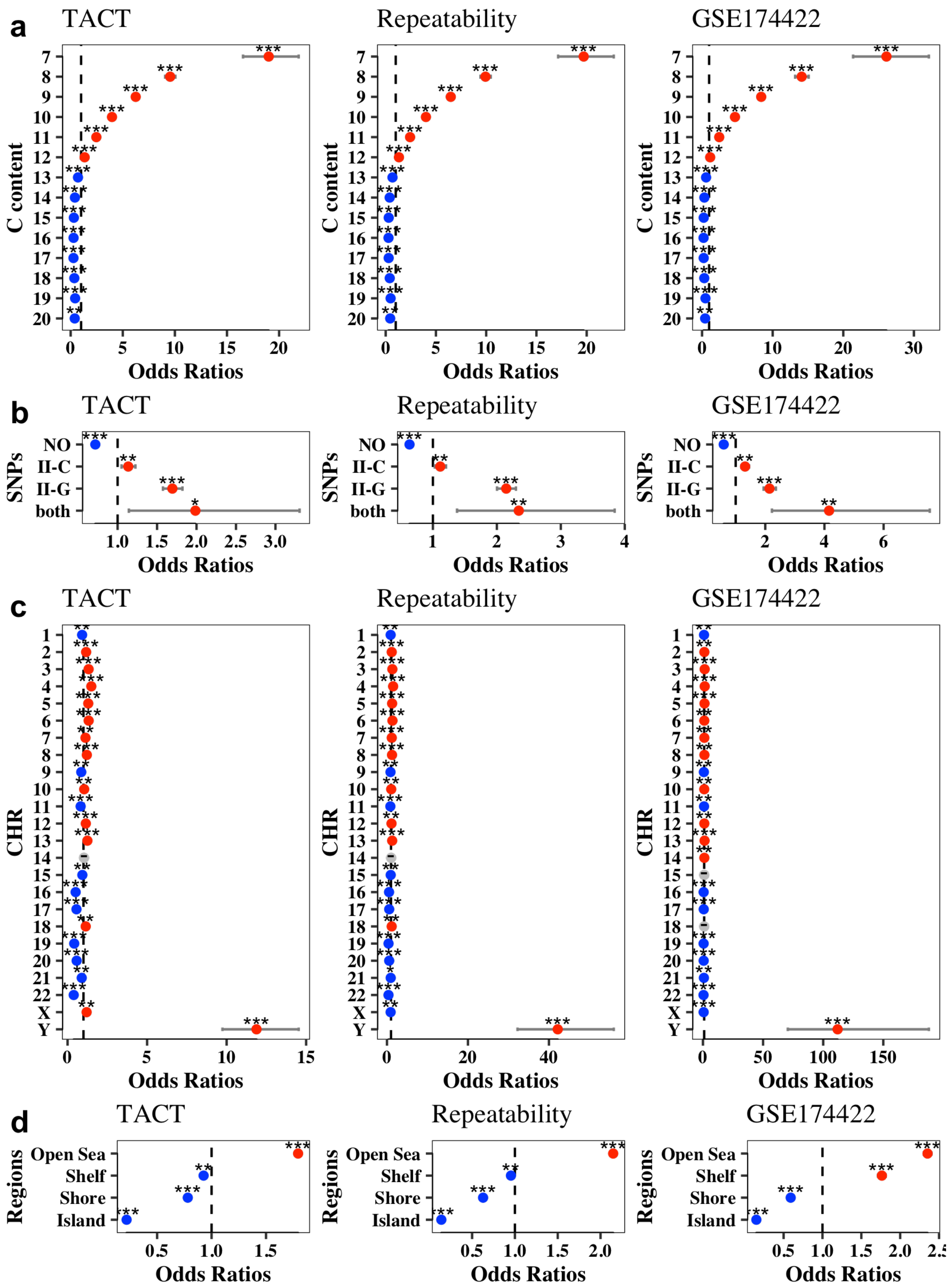

Figure S21. C content, probes targeting SNPs, probe target chromosome and CpG context for unreliable Infinium type II probes. Odds ratios and associated p-values were obtained with a two-sided Fisher test. Non-significant (FDR-adjusted p-value > 0.05) results are shown in grey. Significant results (FDR-adjusted p-value ≤ 0.05) with an odds ratio > 1 or ≤ 1 are shown in red and blue, respectively.

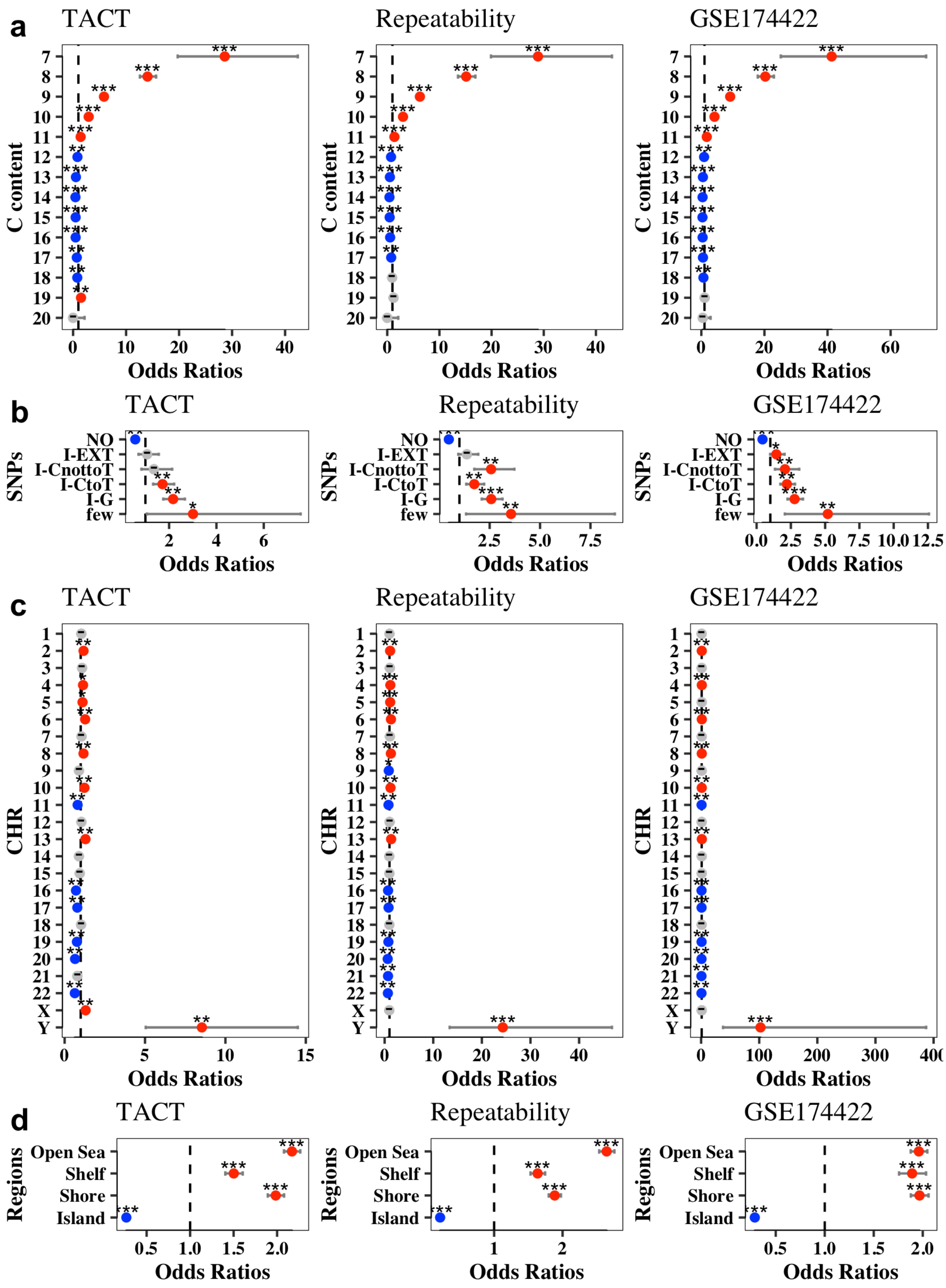

Figure S22. C content, probes targeting SNPs, probe target chromosome and CpG context for unreliable Red Infinium type I probes. Odds ratios and associated p-values were obtained with a two-sided Fisher test. Non-significant (FDR-adjusted p-value > 0.05) results are shown in grey. Significant results (FDR-adjusted p-value ≤ 0.05) with an odds ratio > 1 or ≤ 1 are shown in red and blue, respectively.

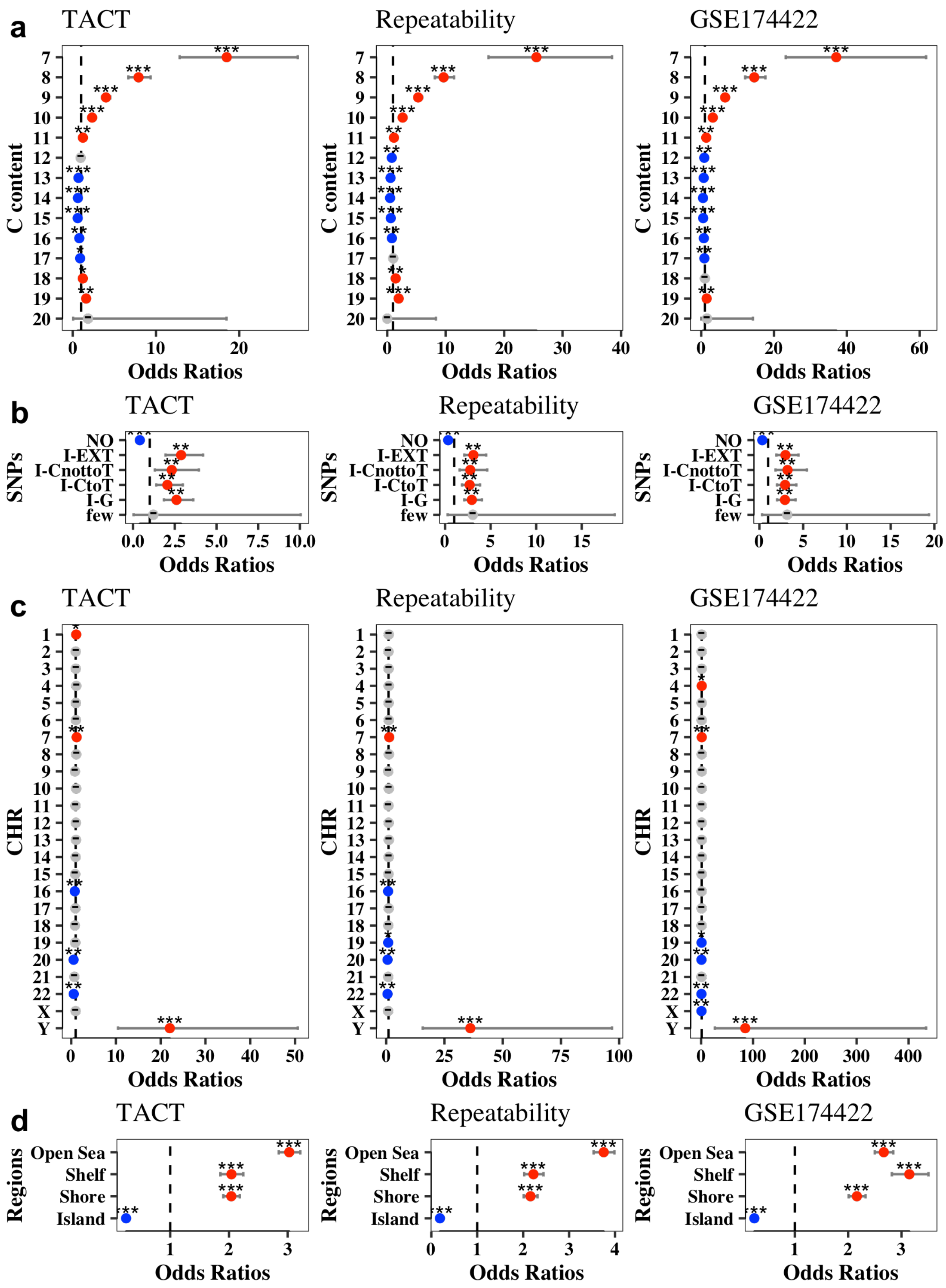

Figure S23. C content, probes targeting SNPs, probe target chromosome and CpG context for unreliable Green Infinium type I probes. Odds ratios and associated p-values were obtained with a two-sided Fisher test. Non-significant (FDR-adjusted p-value > 0.05) results are shown in grey. Significant results (FDR-adjusted p-value ≤ 0.05) with an odds ratio > 1 or ≤ 1 are shown in red and blue, respectively.

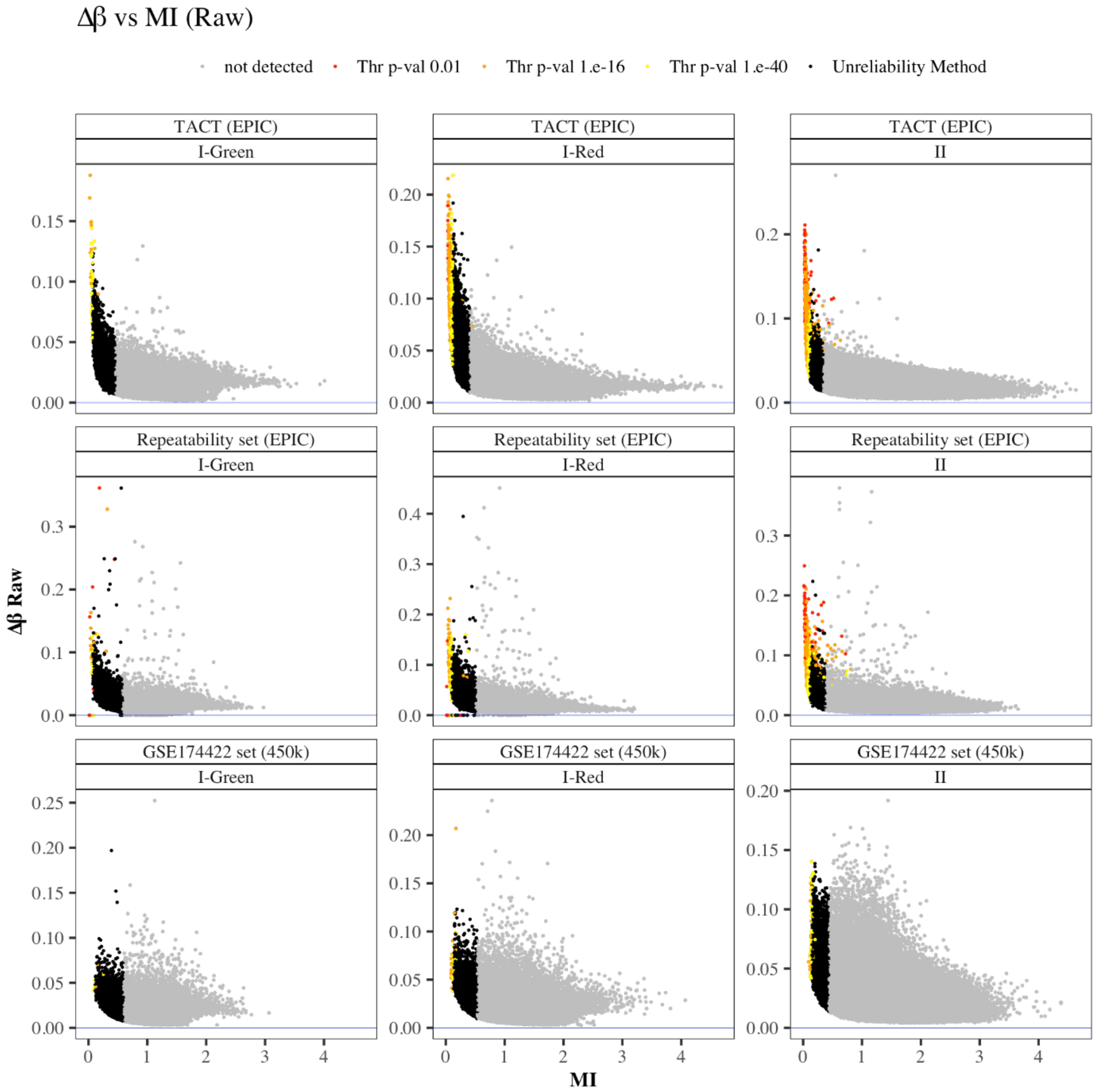

Figure S24. Dependence of Δβ on mean intensity (MI) in the three different datasets. Colors show unreliable probes determined with the detP method using different thresholds (red, orange, yellow) and the Unreliability Method (black). β-values were not normalized.

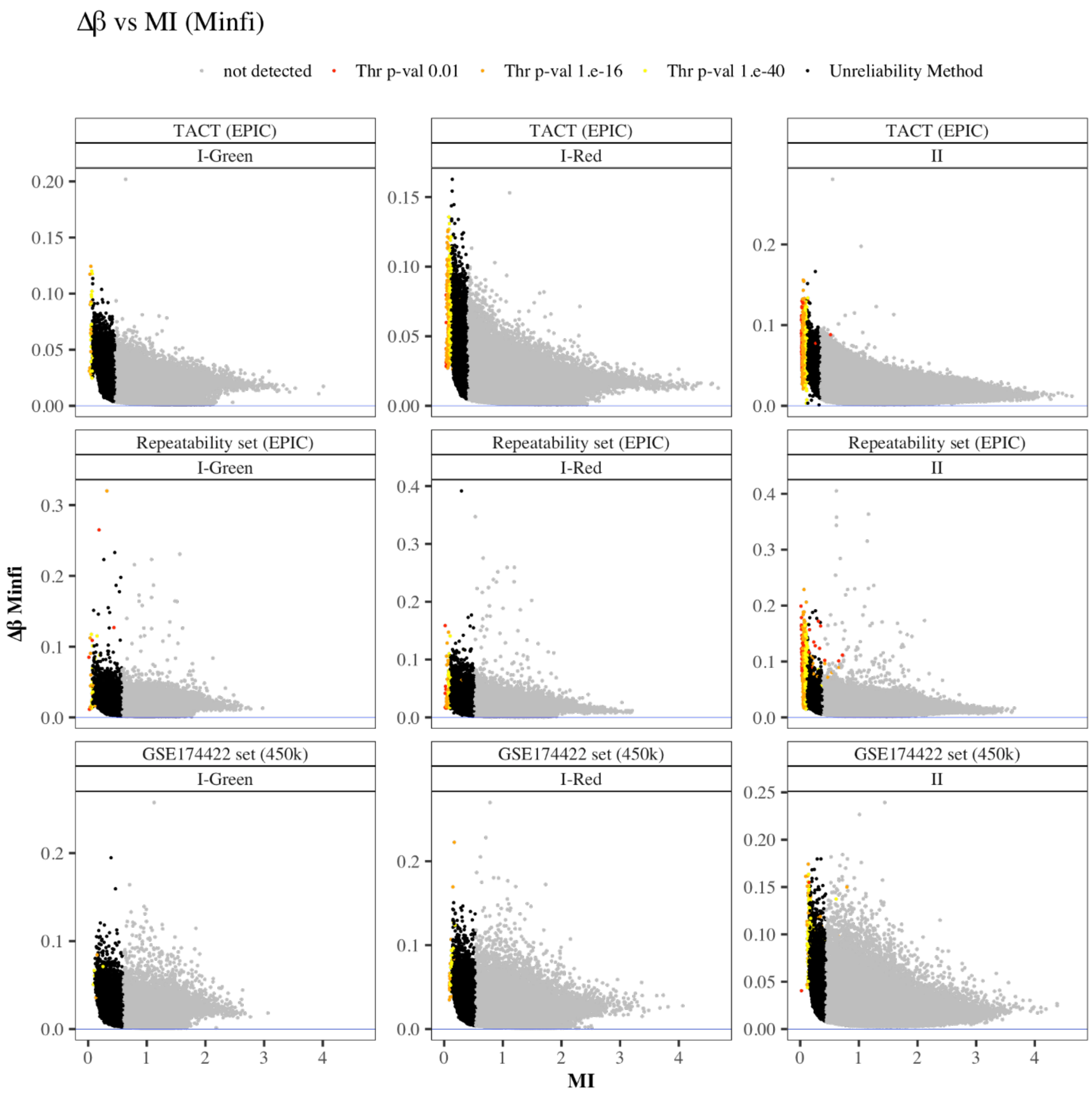

Figure S25. Dependence of Δβ on mean intensity (MI) in the three different datasets. Colors show unreliable probes determined with the detP method using different thresholds (red, orange, yellow) and the Unreliability Method (black). β-values were normalized using the preprocessFunnorm() function in the R package *minfi*.

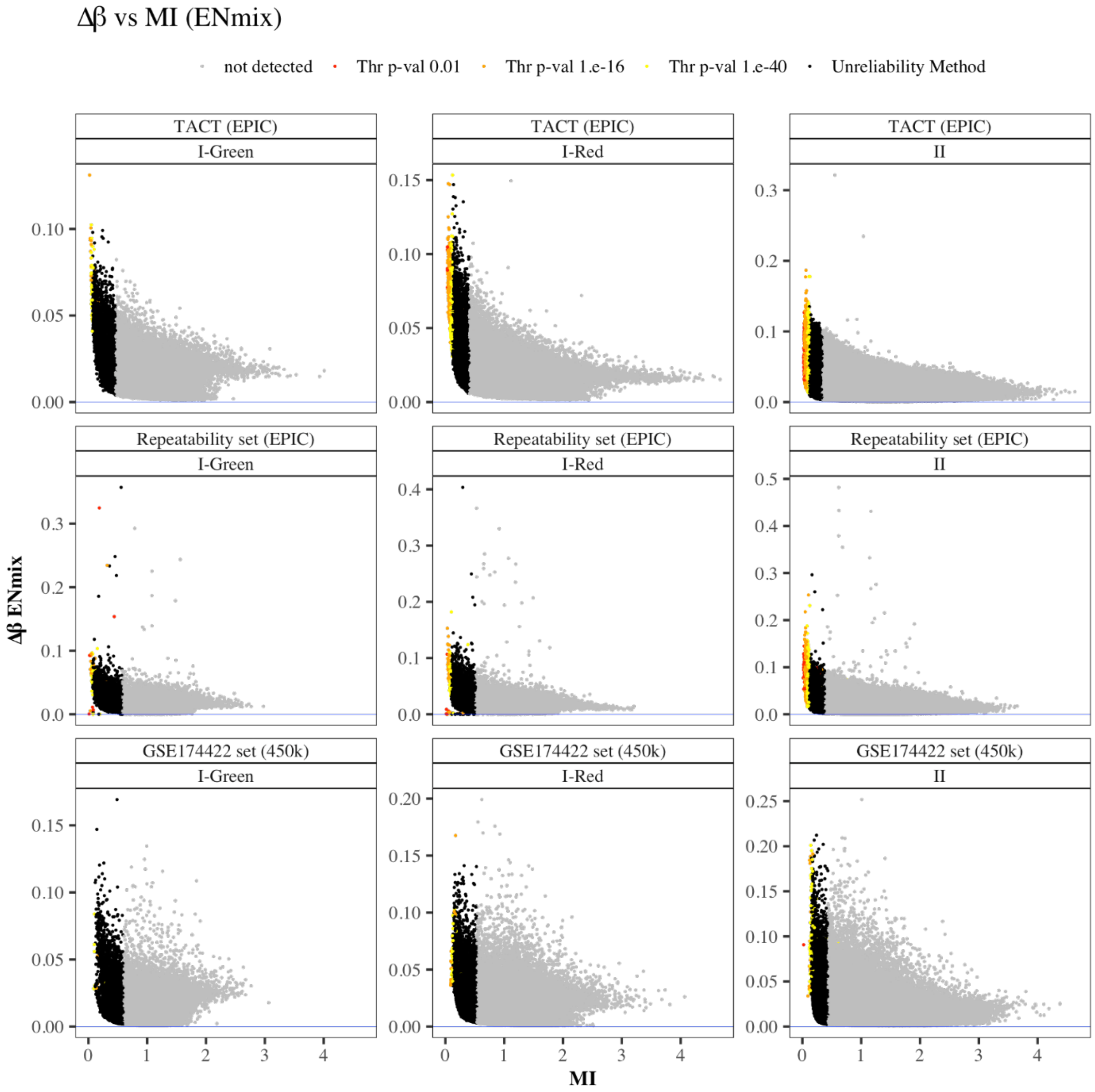

Figure S26. Dependence of Δβ on mean intensity (MI) in the three different datasets. Colors show unreliable probes determined with the detP method using different thresholds (red, orange, yellow) and the Unreliability Method (black). β-values were normalized using the R package *ENmix*.

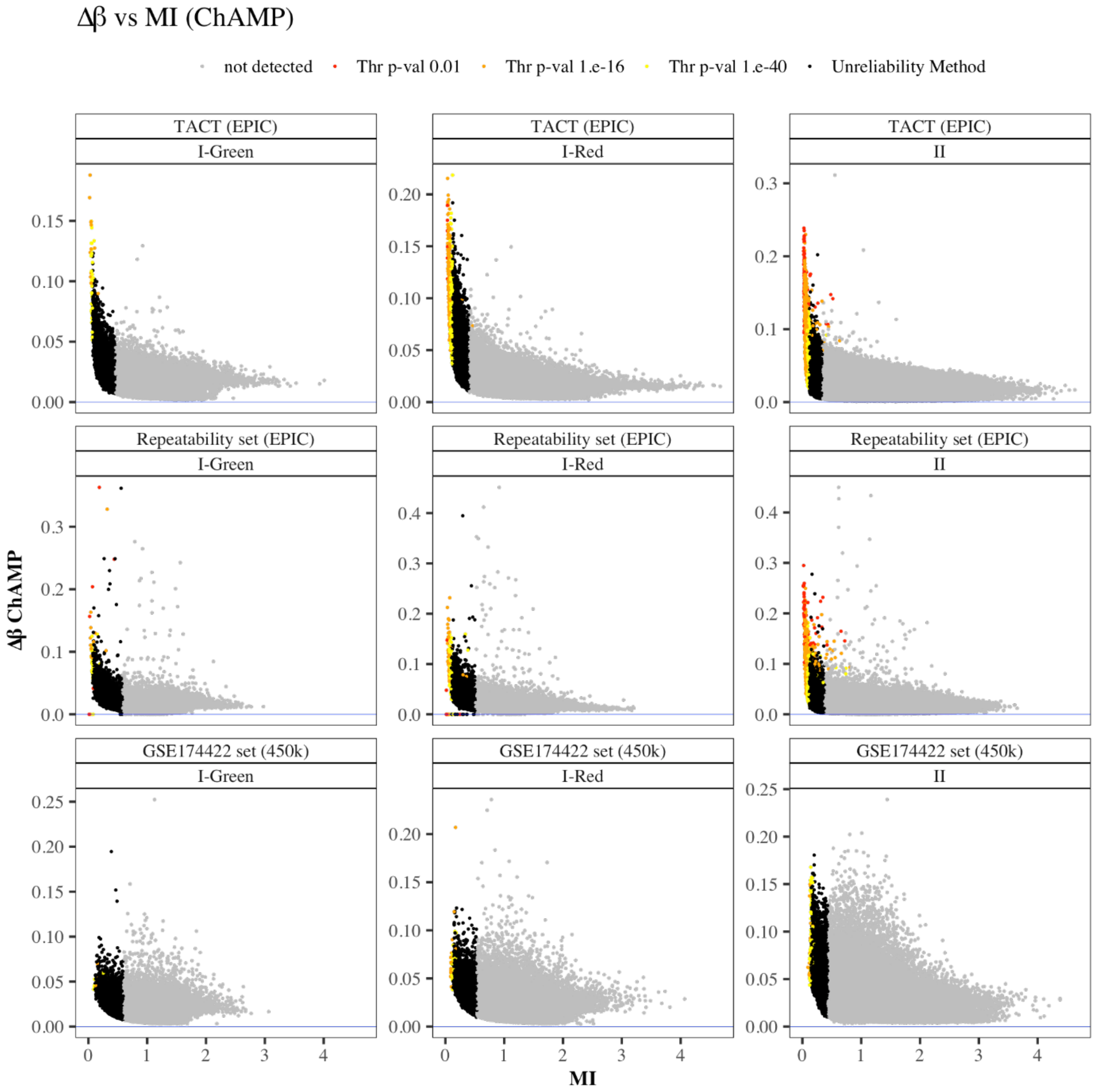

Figure S27. Dependence of Δβ on mean intensity (MI) in the three different datasets. Colors show unreliable probes determined with the detP method using different thresholds (red, orange, yellow) and the Unreliability Method (black). β-values were normalized using the R package *ChAMP*.

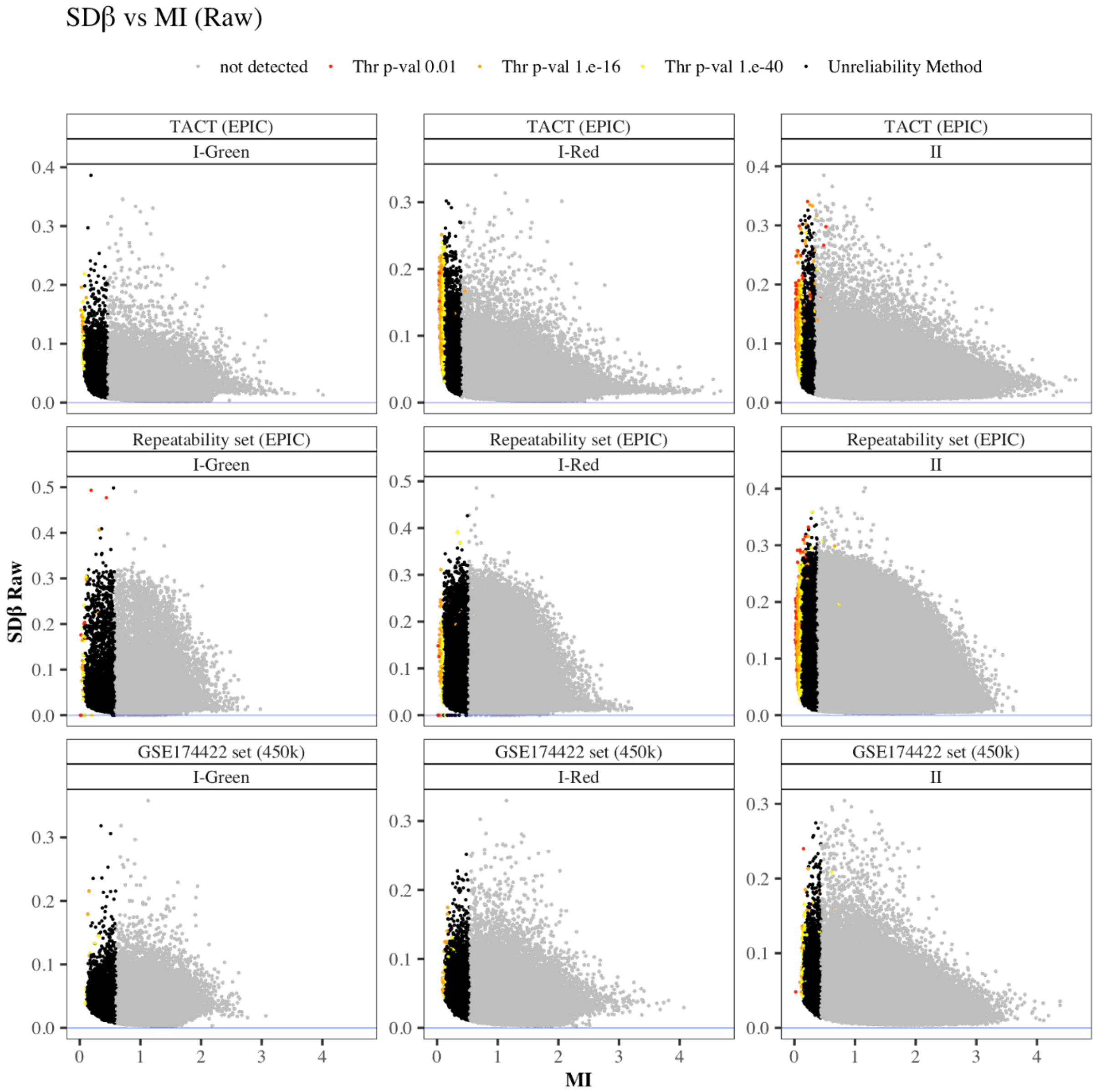

Figure S28. Dependence of SDβ on mean intensity (MI) in the three different datasets. Colors show unreliable probes determined with the detP method using different thresholds (red, orange, yellow) and the Unreliability Method (black). β-values were not normalized.

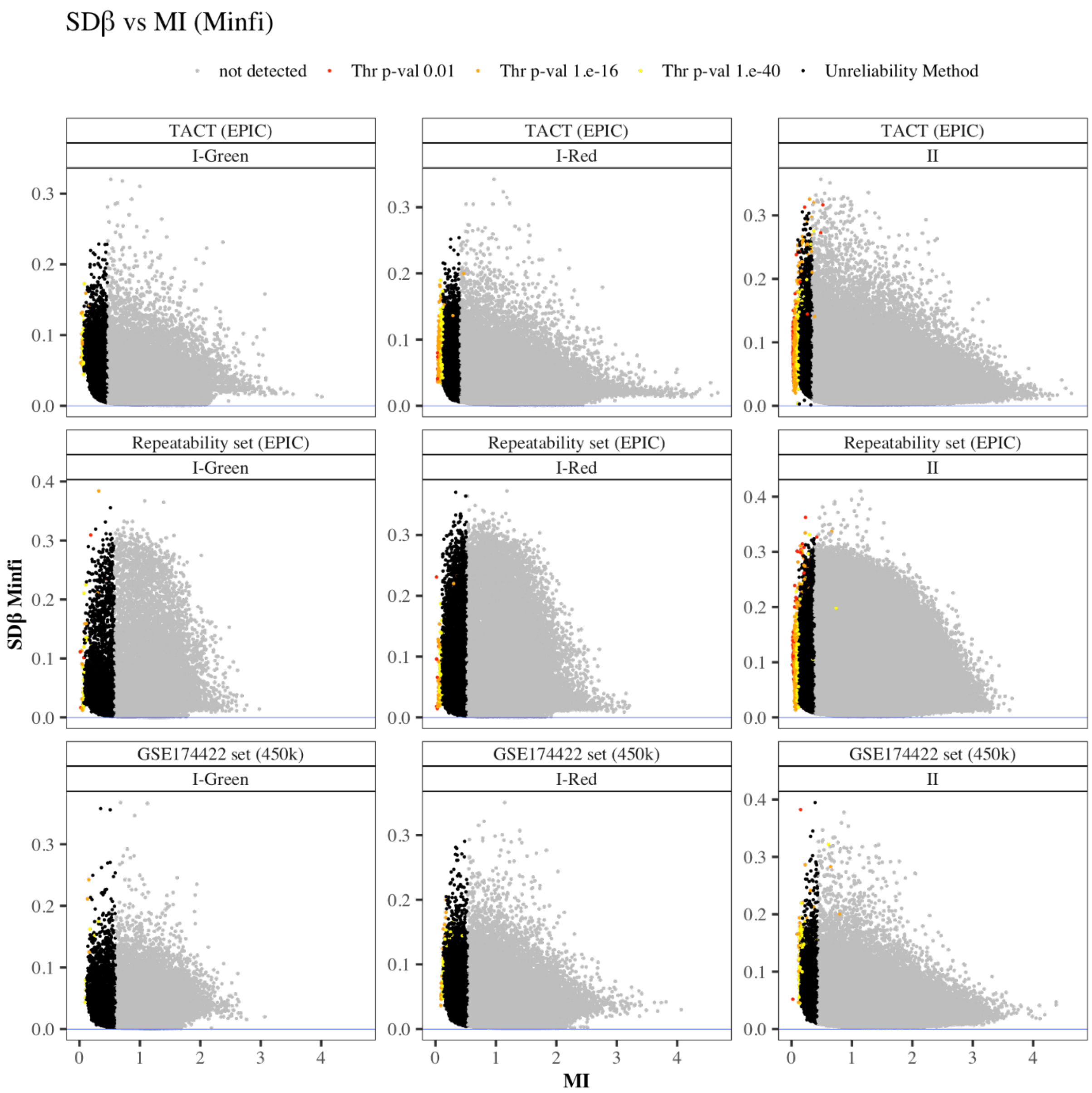

Figure S19. Dependence of SDβ on mean intensity (MI) in the three different datasets. Colors show unreliable probes determined with the detP method using different thresholds (red, orange, yellow) and the Unreliability Method (black). β-values were normalized using the preprocessFunnorm() function in the R package *minfi*.

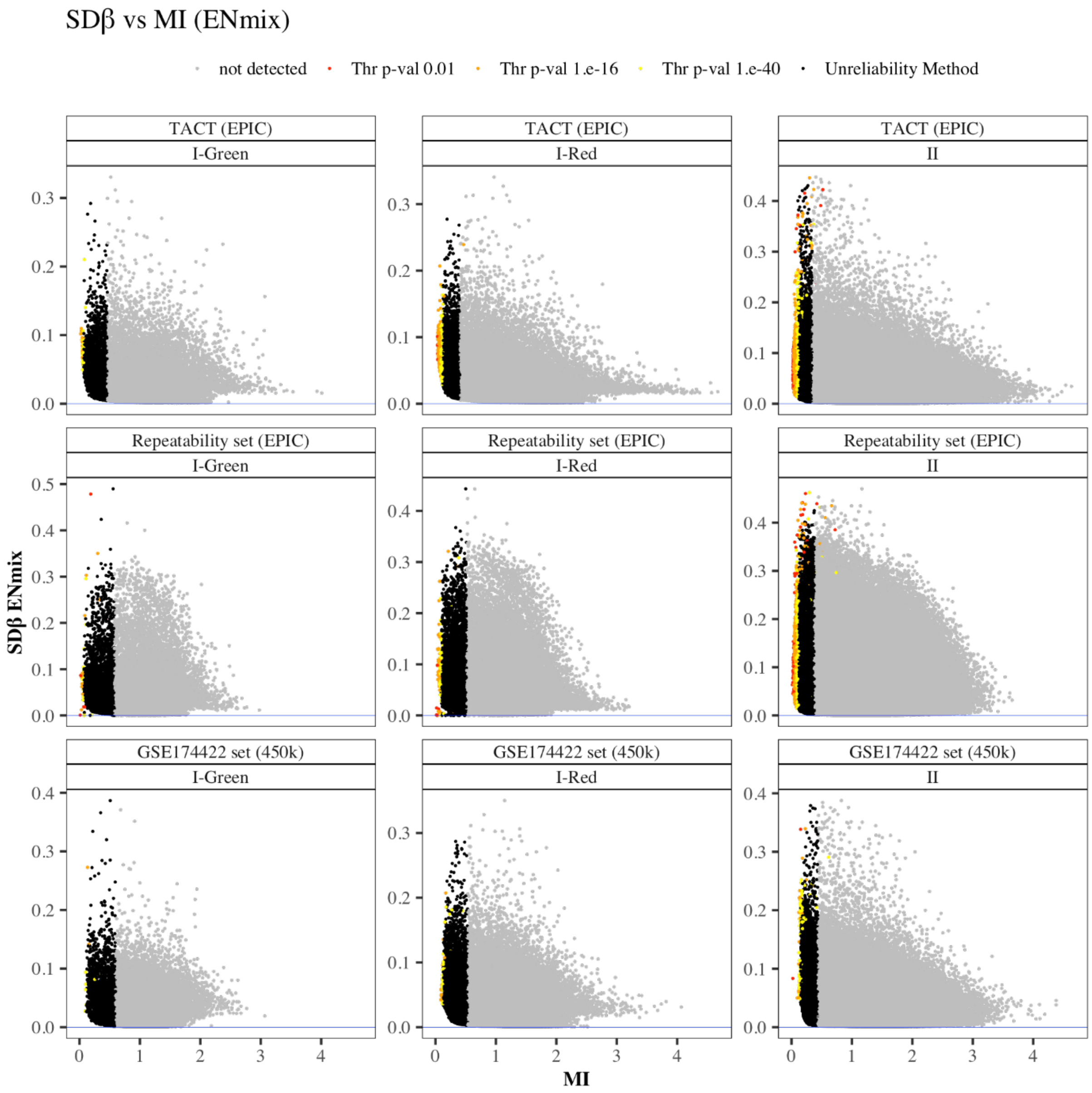

Figure S30. Dependence of SDβ on mean intensity (MI) in the three different datasets. Colors show unreliable probes determined with the detP method using different thresholds (red, orange, yellow) and the Unreliability Method (black). β-values were normalized using the R package *ENmix*.

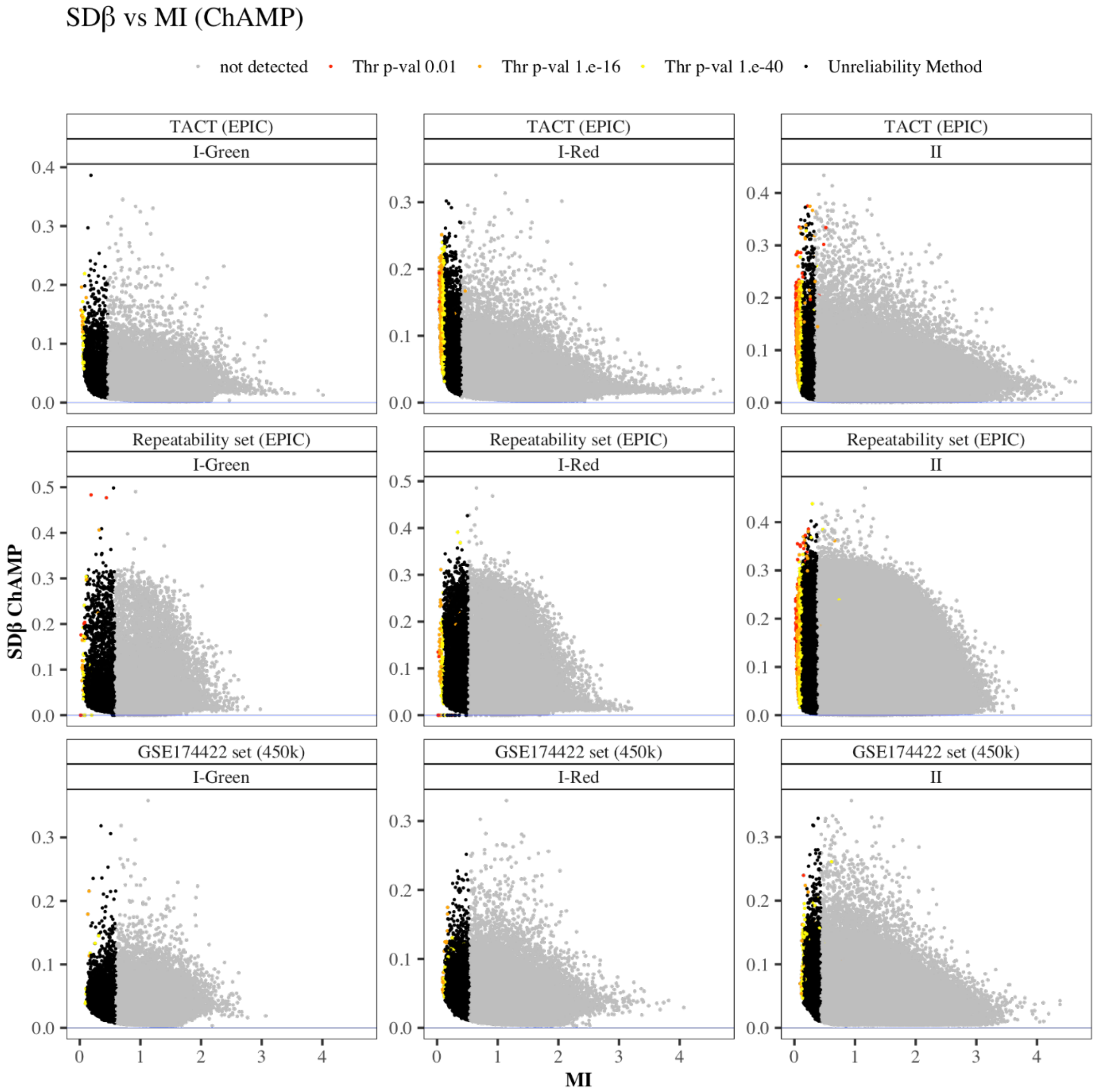

Figure S31. Dependence of SDβ on mean intensity (MI) in the three different datasets. Colors show unreliable probes determined with the detP method using different thresholds (red, orange, yellow) and the Unreliability Method (black). β-values were normalized using the R package *ChAMP*.

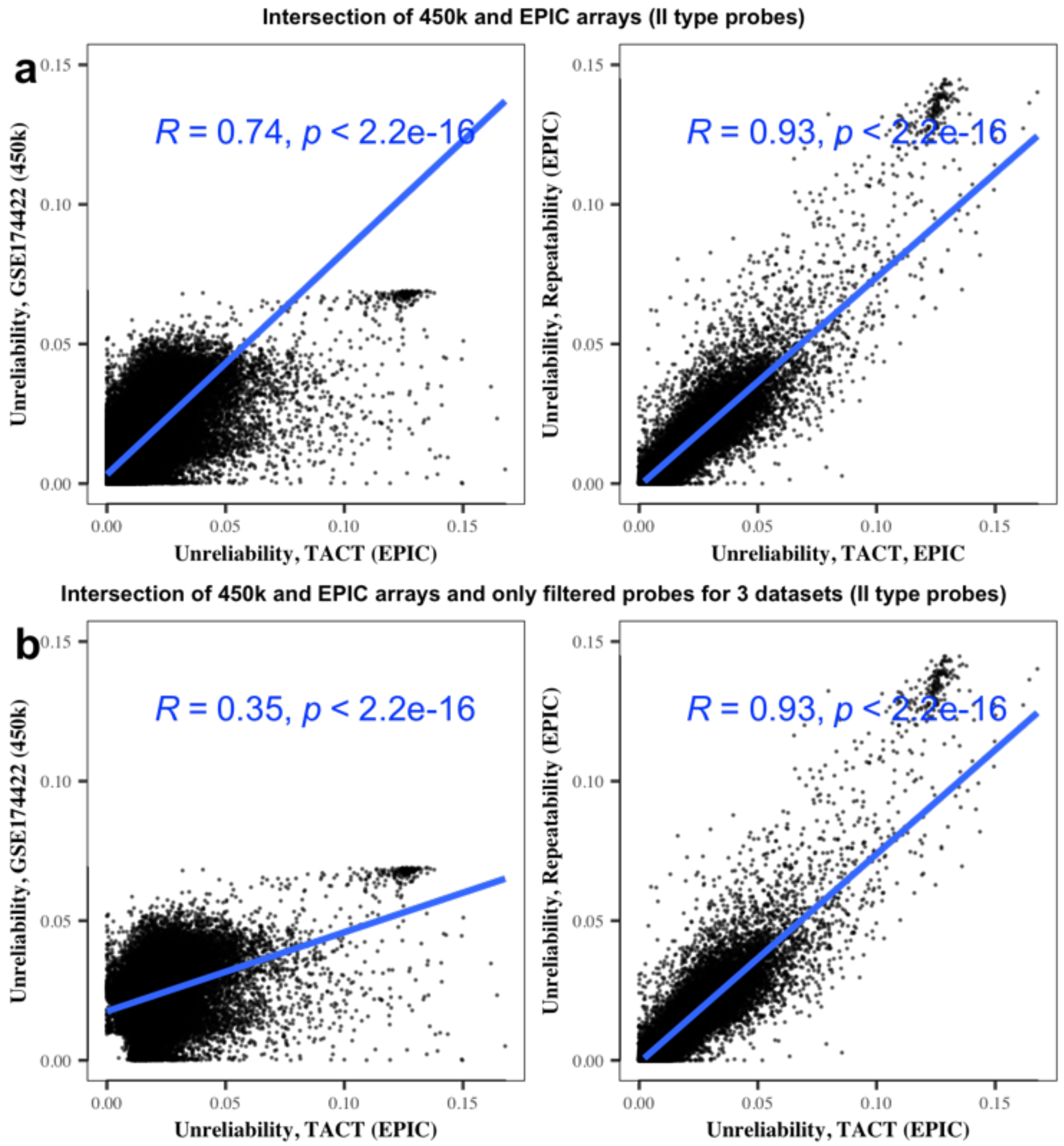

Figure S32. Correlation of Unreliability scores between different datasets on type II probes. (a) Correlation between Unreliability scores of GSE174422 and TACT (left panel) and Repeatability and TACT (right panel) datasets on type II probes in intersection of 450k and EPIC arrays. **(b)** Correlation between Unreliability scores of GSE174422 and TACT (left panel) and Repeatability and TACT (right panel) datasets only on filtered type II probes (intersection of 3 datasets) in intersection of 450k and EPIC arrays.

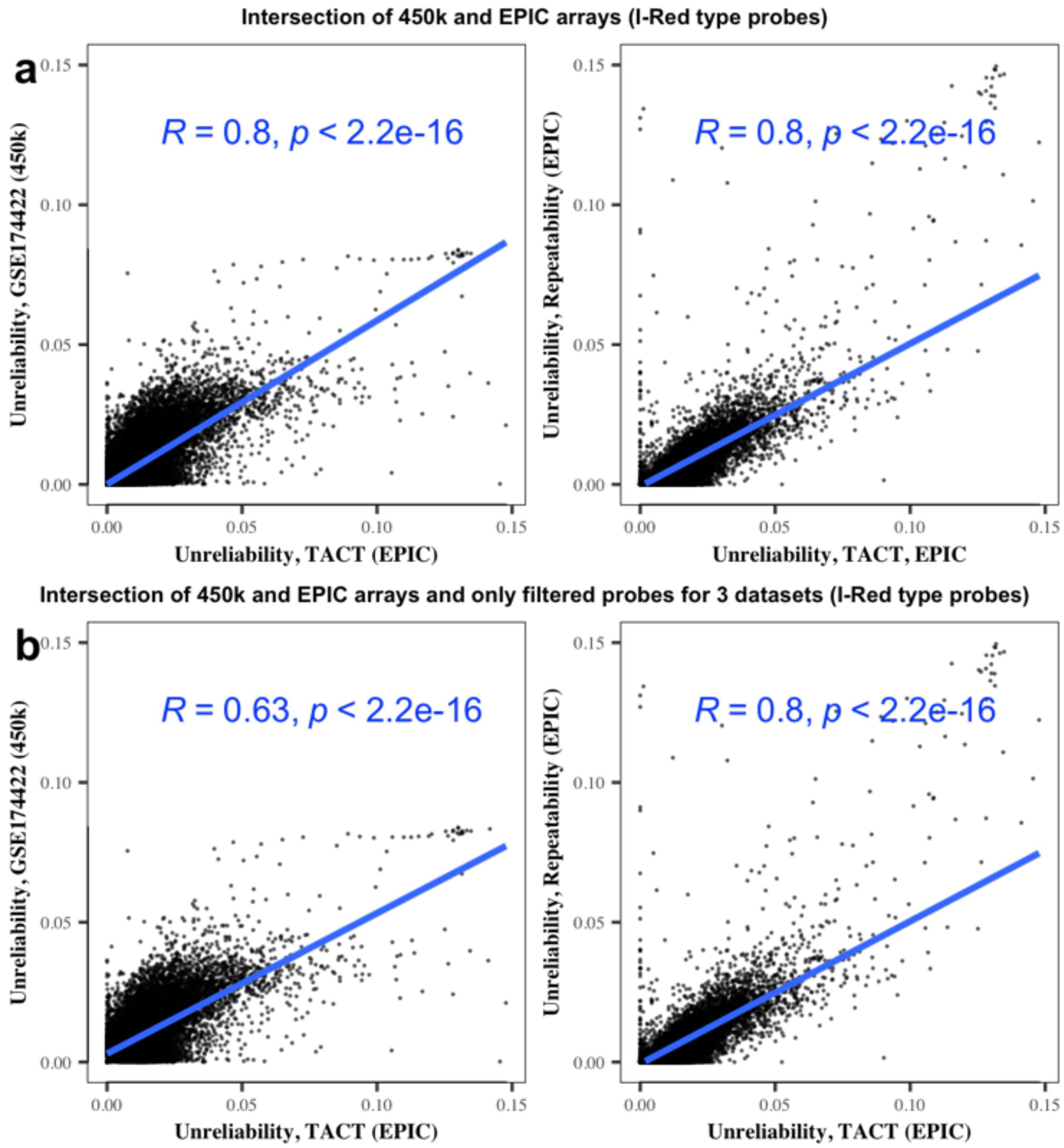

Figure S33. Correlation of Unreliability scores between different datasets on type I-Red probes. **(a)** Correlation between Unreliability scores of GSE174422 and TACT (left panel) and Repeatability and TACT (right panel) datasets on type I-Red probes in intersection of 450k and EPIC arrays. **(b)** Correlation between Unreliability scores of GSE174422 and TACT (left panel) and Repeatability and TACT (right panel) datasets only on filtered type I-Red probes (intersection of 3 datasets) in intersection of 450k and EPIC arrays.

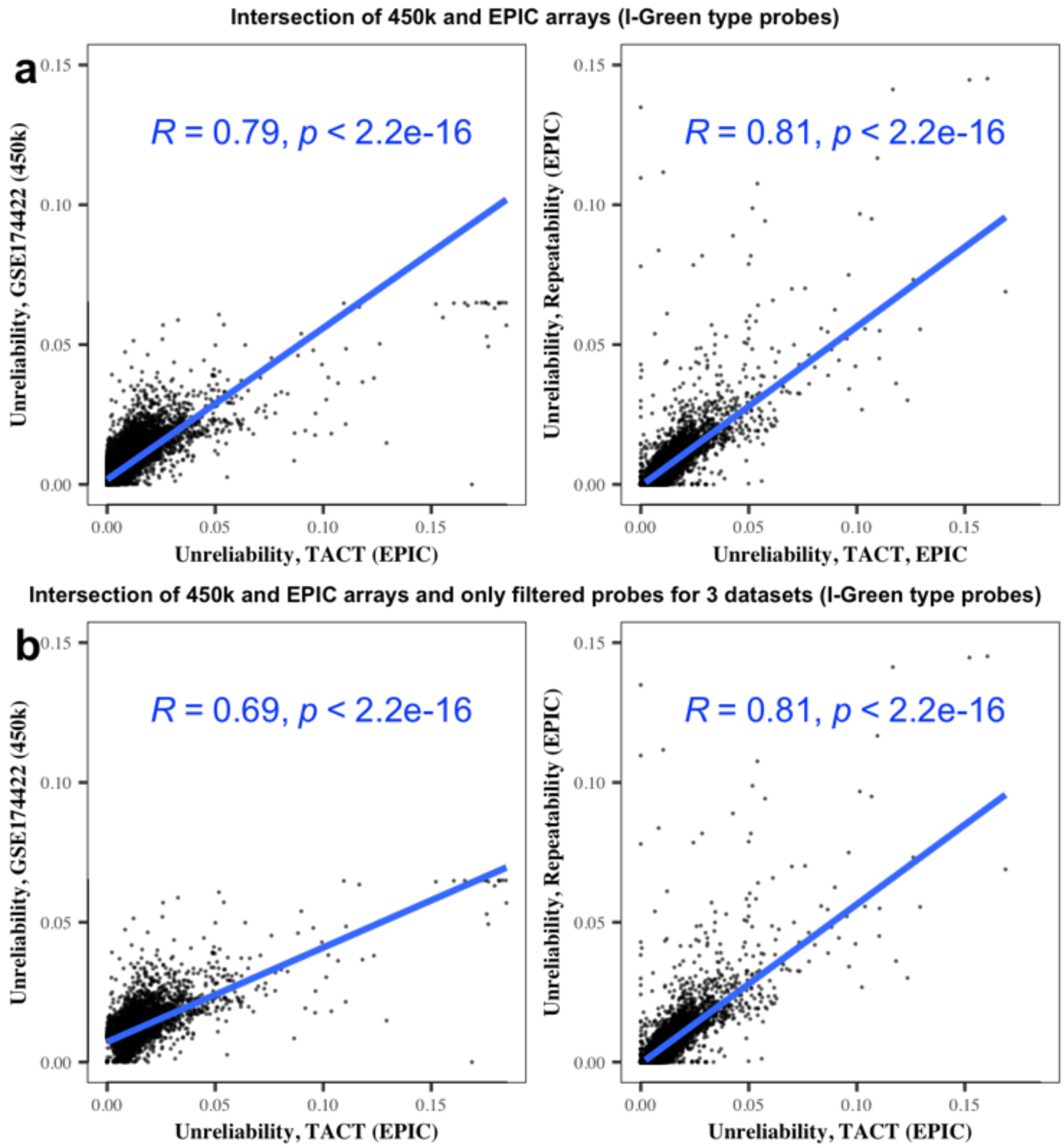

Figure S34. Correlation of Unreliability scores between different datasets on type I-Green probes. (a) Correlation between Unreliability scores of GSE174422 and TACT (left panel) and Repeatability and TACT (right panel) datasets on type I-Green probes in intersection of 450k and EPIC arrays. **(b)** Correlation between Unreliability scores of GSE174422 and TACT (left panel) and Repeatability and TACT (right panel) datasets only on filtered type I-Green probes (intersection of 3 datasets) in intersection of 450k and EPIC arrays.

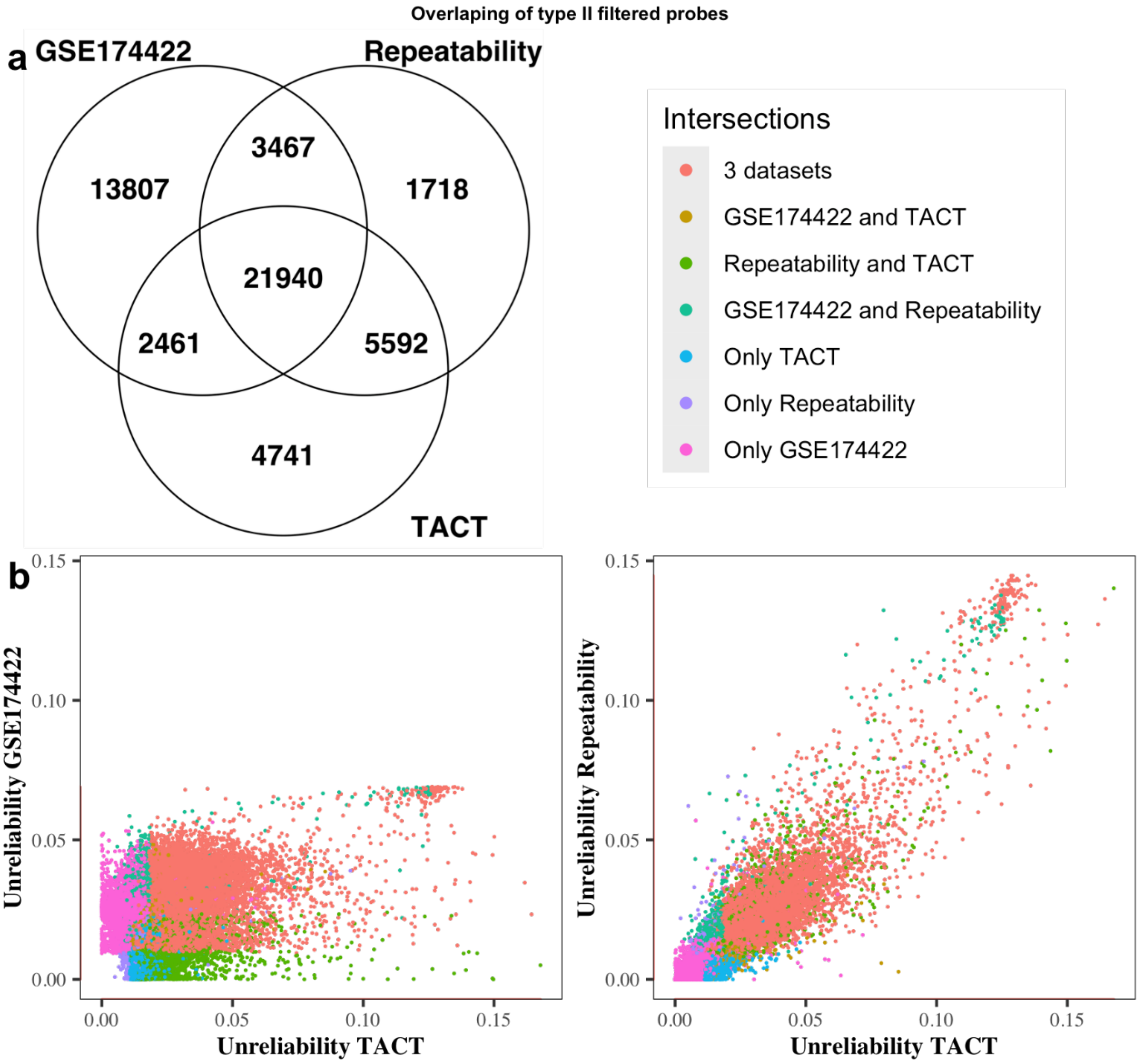

Figure S35. Overlapping and unique unreliable Infinium type II probes in three different datasets.

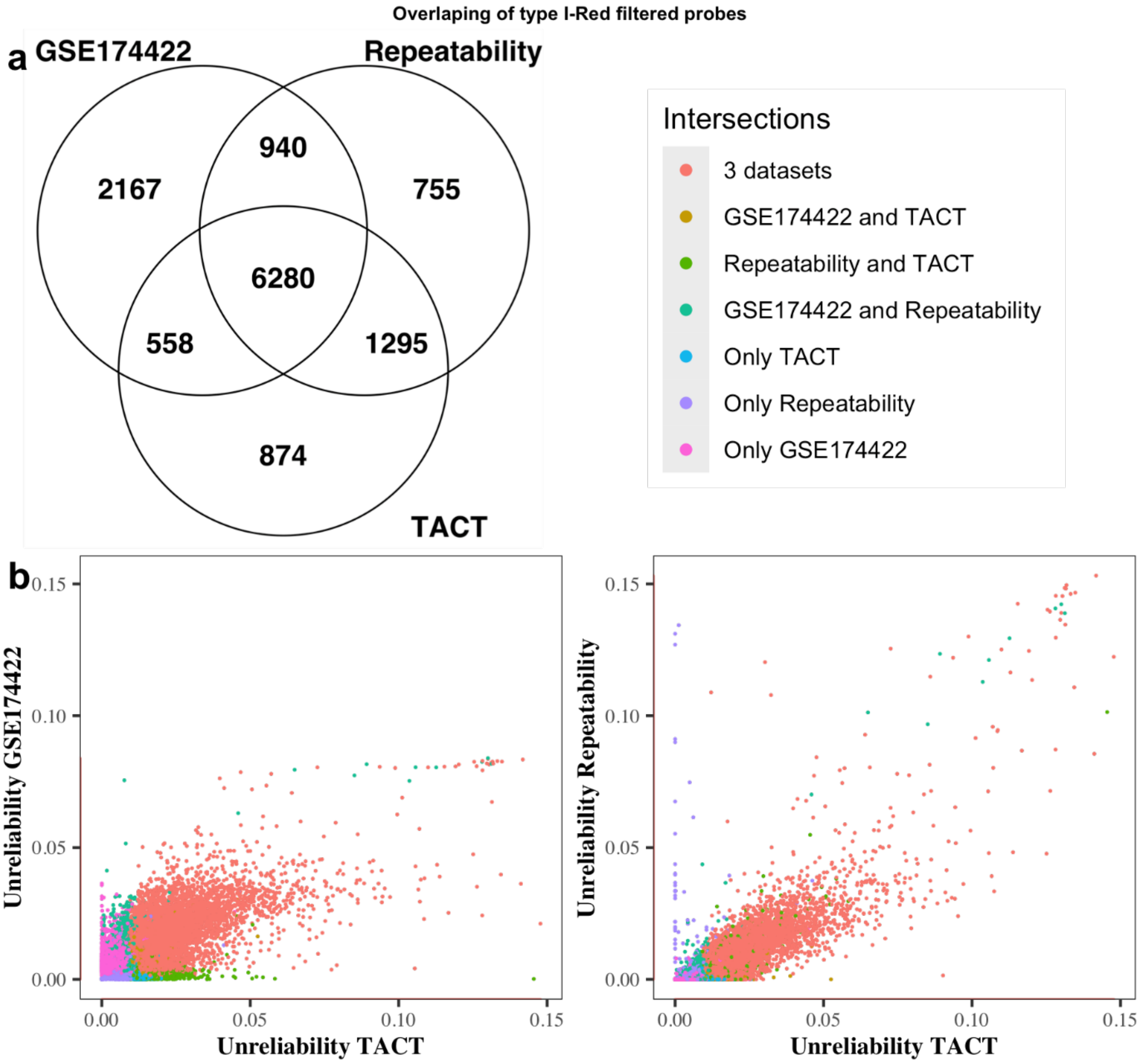

Figure S36. Overlapping and unique unreliable Red Infinium type I probes in three different datasets.

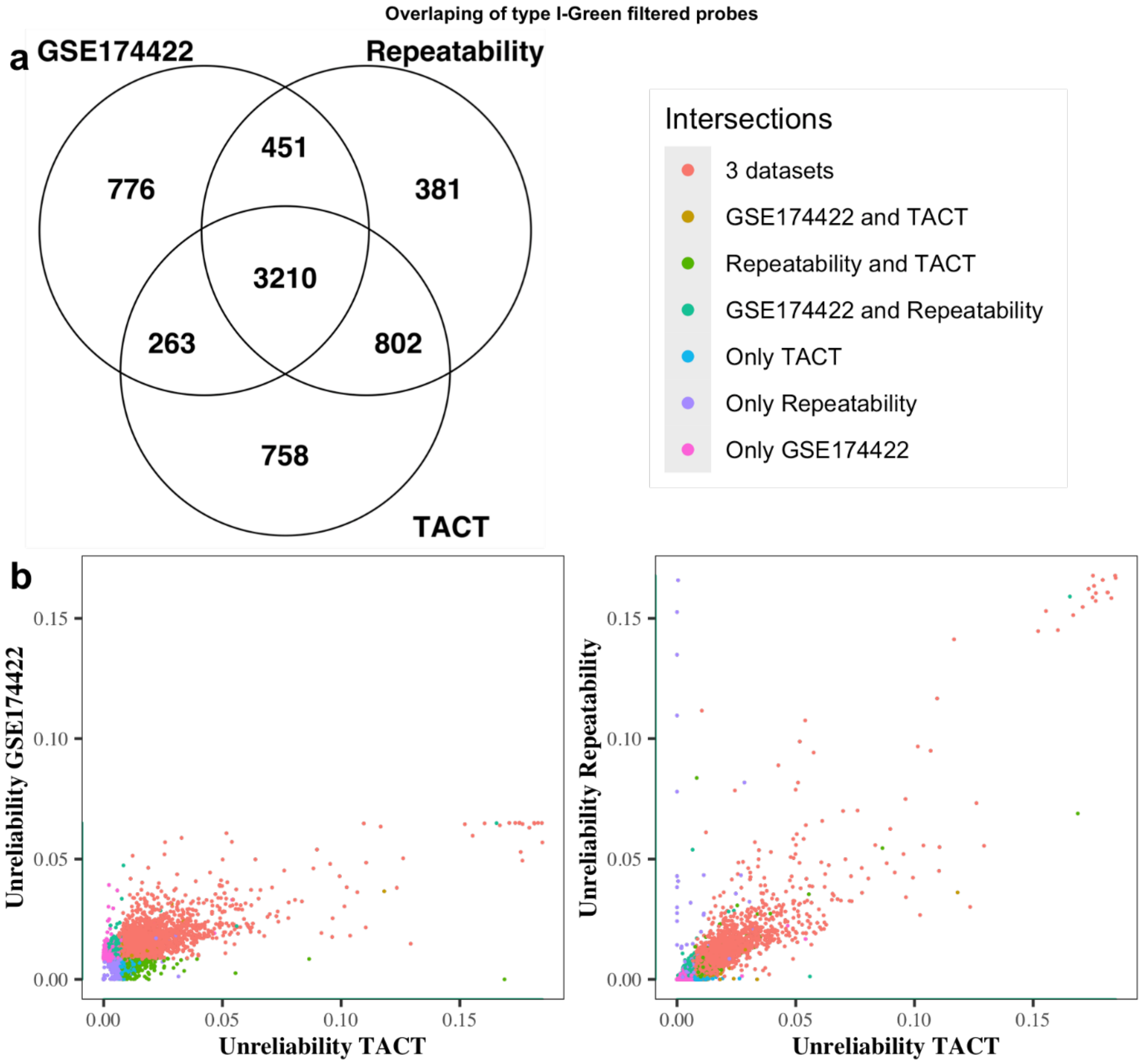

Figure S37. Overlapping and unique unreliable Green Infinium type I probes in three different datasets.

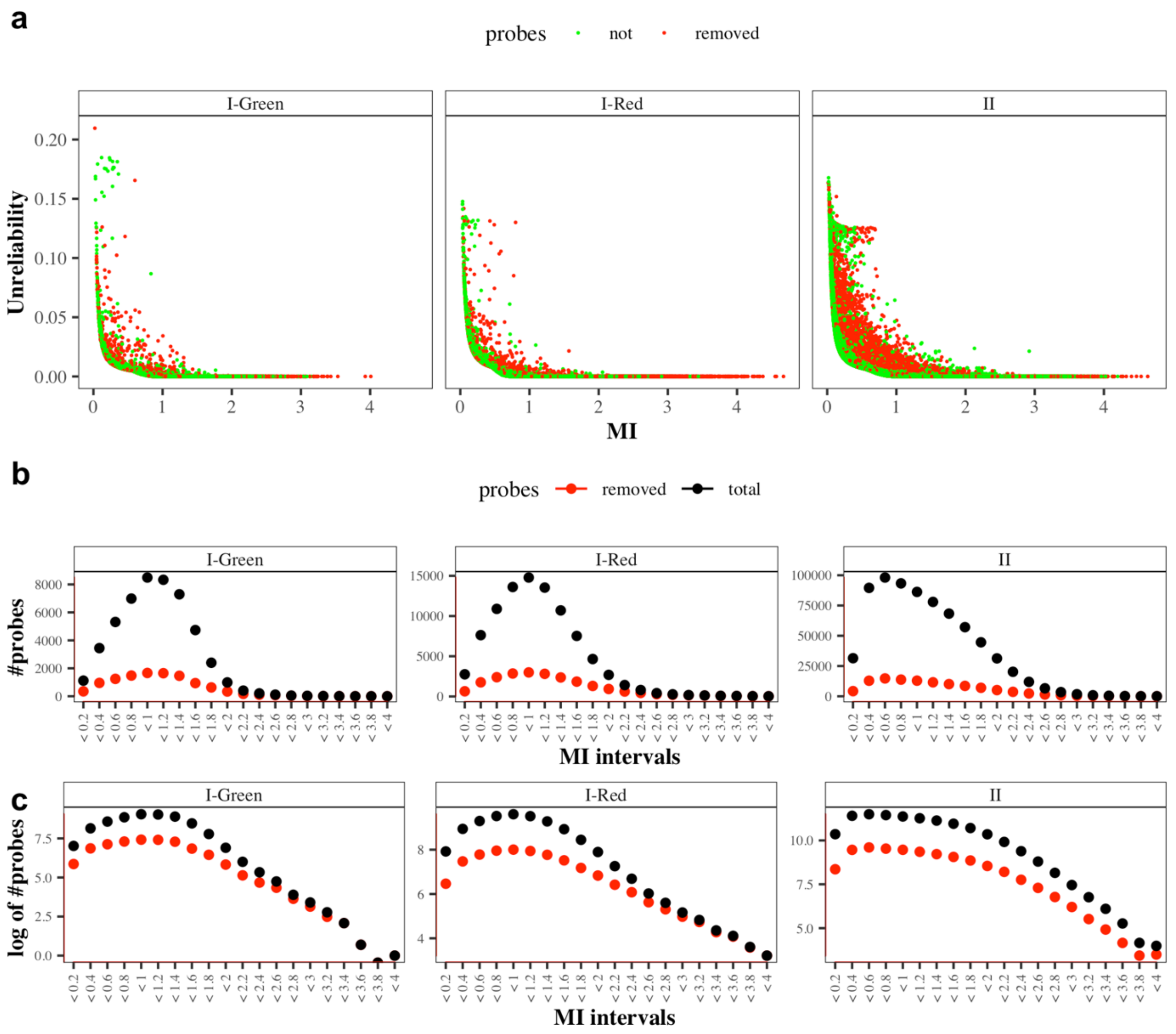

Figure S38. Probes on MethylationEPIC BeadChip v2.0 retained or removed from v1.0. to with the level of their average normalized intensities (MI). (a) The removed probes (red dots) do not tend to be low intensity probes and the overall trend for the remaining probes (green dots) still shows tendency to have high unreliability scores on low intensities levels. There is no bias towards the removal of probes with low intensity both in terms of the number of removed probes relative to **(b)** the total number of probes or **(c)** the logarithmic values of the number of probes.

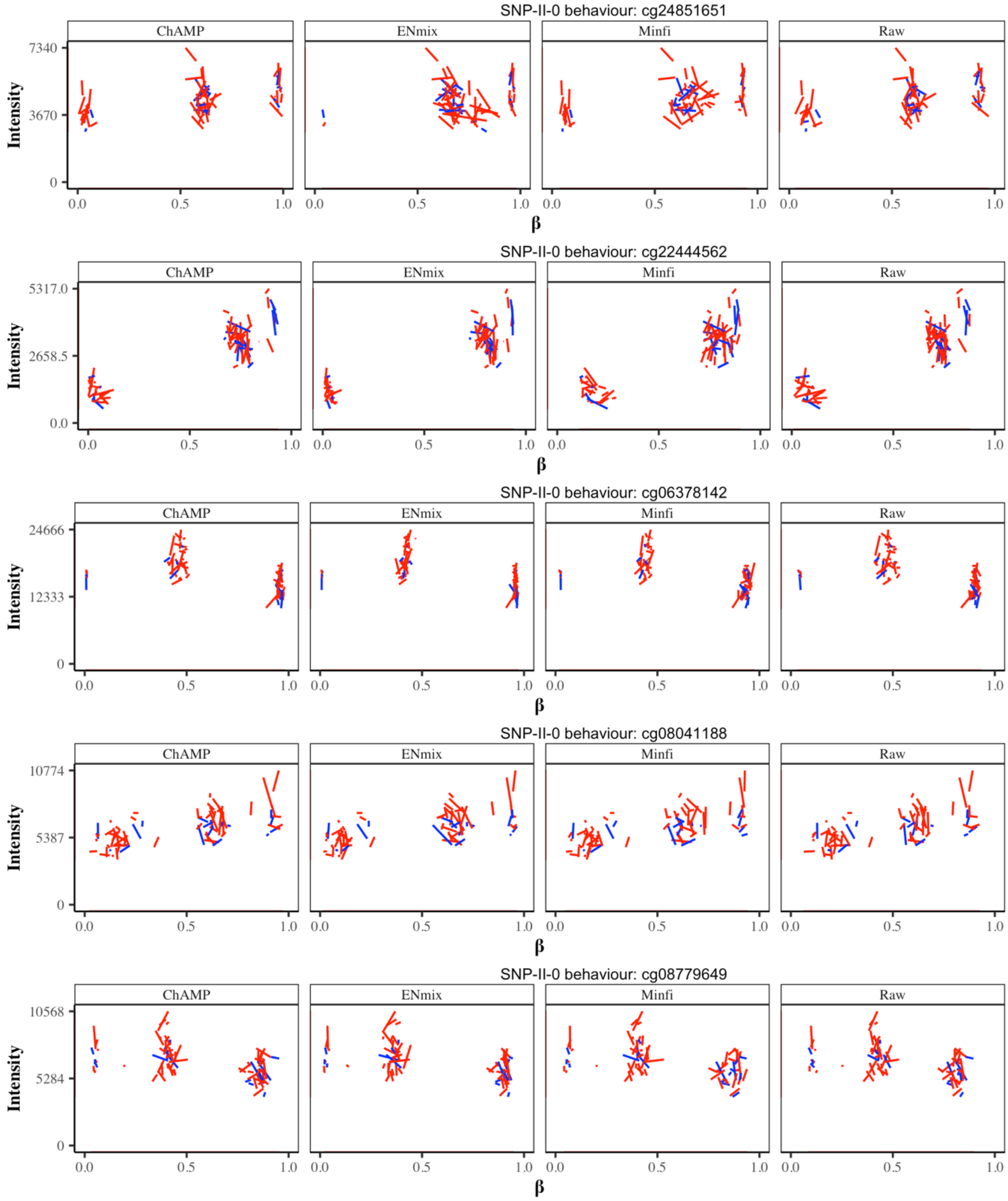

Figure S39. Examples of type II probes retained on MethylationEPIC BeadChip v2.0 from v1.0 exhibiting SNP-II-0 behavior, but not marked in the manifest. For clarification on SNP-II-0 behaviour see Figure 1d (main text) and Supplementary Figure_old S1.

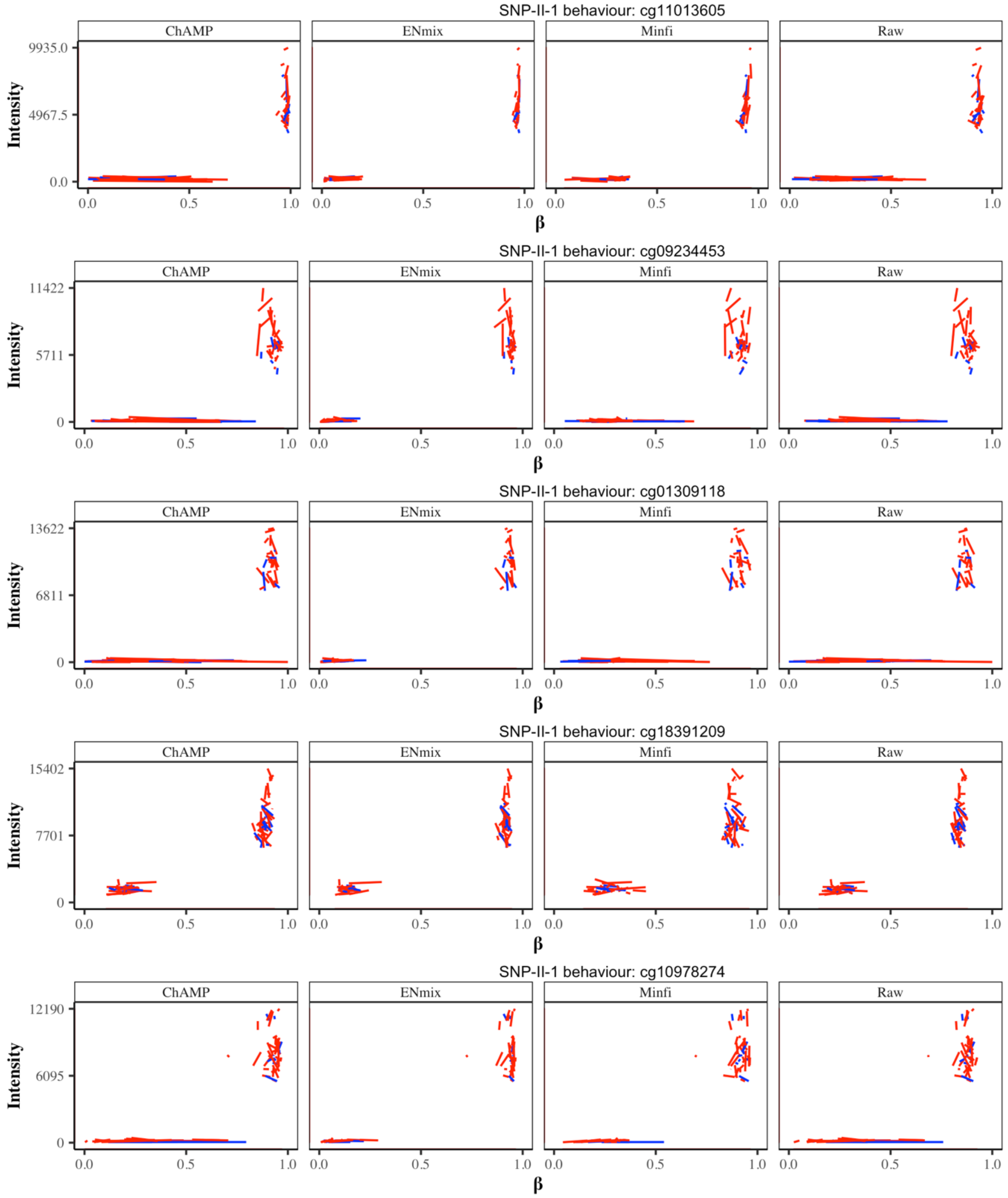

Figure S40. ***Examples of type II probes retained on MethylationEPIC BeadChip v2.0 from v1.0 exhibiting SNP-II-1 behavior, but not marked in the manifest.*** *For clarification on SNP-II-1 behaviour see Figure 1d (main text).*
